# Self-illuminating quantum dots for non-invasive bioluminescence imaging of mammalian gametes

**DOI:** 10.1186/s12951-015-0097-1

**Published:** 2015-06-04

**Authors:** Jean M Feugang, Ramey C Youngblood, Jonathan M Greene, Scott T Willard, Peter L Ryan

**Affiliations:** Department of Animal and Dairy Sciences, Facility for Organismal and Cellular Imaging (FOCI), Mississippi State University, Mississippi State, MS 39762 USA; Department of Pathobiology and Population Medicine, Mississippi State University, Mississippi State, MS 39762 USA; Department of Biochemistry and Molecular Biology and Entomology and Plant Pathology, Mississippi State University, Mississippi State, MS 39762 USA

**Keywords:** Bio-imaging, Bio-sensing, Follicle, Nanoparticles, Oocyte, Plasminogen, Spermatozoa

## Abstract

**Background:**

The fertility performance of animals is still a mystery and the full comprehension of mammalian gametes maturation and early embryonic development remains to be elucidated. The recent development in nanotechnology offers a new opportunity for real-time study of reproductive cells in their physiological environments. As a first step toward that goal, we evaluated the effectiveness of a fluorescent and luminescent nanoparticle for in vitro and ex vivo imaging of porcine gametes.

**Methods:**

Freshly harvested boar sperm were labeled with red-shifted (655 nm) quantum dot nanoparticles conjugated (QD+) or not (QD−) with plasminogen antibody and evaluated. Subsets of labeled spermatozoa were loaded into straws and placed within the lumen of gilt reproductive tracts for ex vivo intra-uterine imaging. Porcine cumulus-oocyte complexes (COCs) were matured in the presence of QD− or QD+. Ovarian follicles were microinjected with QD− or QD+ and placed in culture for up to 4 days. After labeling, all samples were supplemented with coelenterazine, the luciferase substrate, and immediately submitted to bioluminescence analysis, followed by fluorescence and hyperspectral imaging. Data were analyzed with ANOVA and P < 0.05 indicated significant differences.

**Results:**

All labeled-samples revealed bioluminescence emission that was confirmed by fluorescence and hyperspectral imaging of the QD localization within the cells and tissues. Over 76% of spermatozoa and both immature and mature COCs were successfully labeled with QD− or QD+. The QD− fluorescence appeared homogenously distributed in the oocytes, while found in the entire sperm length with a higher accumulation within the mid-piece. Labeled-follicles exhibited a progressive migration of QD nanoparticles within the follicle wall during culture. In contrast, QD+ fluorescence signals appeared condensed and stronger in the follicle cells, sperm head, and sub-plasma membrane area of mature oocytes. Weaker QD+ signals were detected in the cumulus cells. Fluorescence and hyperspectral microscope imaging showed comparable intracellular QD localization. Ex-vivo intra-uterine bioluminescence imaging of labeled spermatozoa revealed stronger signals captured over the oviducts, with uterine body allowing the lowest signal detection.

**Conclusion:**

Findings indicate that conjugated and non-conjugated fluorescent nanoparticles can be used for effective labeling of mammalian gametes for in vitro monitoring and potential in vivo targeted-imaging.

**Electronic supplementary material:**

The online version of this article (doi:10.1186/s12951-015-0097-1) contains supplementary material, which is available to authorized users.

## Background

The successful reproduction in mammals is orchestrated by successive and complex events occurring in tissues that are deeply embedded in the animal body. Thus, the examination of mammalian gametes and embryos has been mainly invasive; however, the recent progress in imaging systems, such as the digital videomicroscopy, allows for non-invasive and real-time investigation of gametes and embryos in their physiological conditions [1]. The combination of these imaging systems with fluorescent dyes has the potential to provide a better understanding of the biological and physiological processes related to successful reproduction. Few studies have attempted non-invasive imaging of spermatozoa [2, 3], using fiber-optic fluorescence imaging based upon the green fluorescence protein that is known to have tremendous brightness and photo-stability limitations for deep-tissue imaging. These challenges can be overcome with fluorescent inorganic nanoparticles [4]. The recent progress in nanotechnology provides a new horizon to unfold the mystery of the multifaceted molecular networks that are associated with oocyte maturation and sperm function [5, 6].

Inorganic semi-conductor quantum dots are size-tunable particles of up to 10 nm in diameter. Their unique optical and electronic properties allow size-dependent emission of photo-stable and bright fluorescence, from ultraviolet to near infra-red [7–10]. Over the past two decades, the specific features of quantum dots have been exploited for bio-imaging in biomedical research, especially by rendering them compatible with biological fluids [11–14]. Thus, the possibility to cross-linking these nanoparticles to various biomolecules (protein, antibody, peptide, DNA, etc.) has made them very attractive tools for non-invasive and real-time bio-imaging through cell labeling, single molecule or cell tracking, and diagnostic and targeted therapy [8, 15–17]. The attachment of proteins such as luciferase makes quantum dots suitable for both bioluminescence and fluorescence imaging [6, 18, 19], with the bioluminescence component serving for routine and rapid laboratory confirmation of labeling and the fluorescence part for deep tissue imaging through specific targets, such as protein [20].

Recently, we used luciferase-conjugated fluorescent quantum dots (BRET-QD) to assess their interactions with boar spermatozoa [5]. The QD-BRET complex is a self-illuminating nanoparticle that emits light under incubation with coelenterazine, the luciferase substrate [6]. This enzymatic reaction generates enough energy to excite the quantum dot core, leading to an intense red-shifted fluorescence emission (655 nm) that is crucial for deep-tissue molecular imaging [21]. Our previous study using this nanoparticle complex was an attempt to build on this dual imaging technology to enhance our comprehension of the complex biological and physiological processes of reproduction through non-invasive and real-time analyses [5]. In that pioneer study, we found that, when properly used, BRET-QD nanoparticles interact with spermatozoa without impairing their motility, quality (integrity of plasma and mitochondrial membranes), and viability (ability to fertilize the oocytes) characteristics.

In the present study, we expanded the potential use of QD-BRET nanoparticles for non-invasive imaging of spermatozoa within the female genital tract, while exploring the possibility for imaging cultured ovarian follicles and in vitro matured oocytes. In parallel, we conducted a targeted bio-imaging using the QD-BRET tagged with anti-porcine plasminogen antibody. In previous studies, this protein has been detected in the oocyte and has putative role during fertilization [22–24].

## Methods

### Materials and reagents

Stock solutions of coelenterazine (0.5 mg/ml methanol) and CdSe/ZnS core-shell structure quantum dots (500 nM in Tris buffer) cross-linked to Renilla luciferase (BRET) and nona-arginine R9 peptide (QD-BRET) were purchased from Zymera Inc. (San Jose, CA, USA). Functionalized QD-BRET complexes with the anti-porcine plasminogen antibody (PLG; cat#BP750, Acris Antibodies, Inc., San Diego, CA, USA) were prepared and purchased from Zymera. The functionalization did not affect the QD-BRET fluorescence and both functionalized (QD+) and non-functionalized (QD−) nanoparticles were used in the study. Additional, the anti-human plasminogen antibody was purchased from Santa Cruz Biotechnology (cat#25546; Santa Clara, CA, USA) for confirmation of the functionalized-QD-BRET labeling. Boar semen was purchased at a commercial boar stud (Prestage Farms; West Point, MS, USA) and reproductive tracts with pre-ovulatory ovaries were collected from post-mortem gilts. Fresh samples were transported to the laboratory for the purification of living spermatozoa or the collection of follicles and COCs. Otherwise indicated, all other reagents were purchased from Sigma-Aldrich (Saint-Louis, MO, USA). Washing medium consisted of a pre-warmed PBS supplemented with 1 mg/ml of PVP.

### Evaluation of synthesized QD-BRET nanoparticles

Three options were chosen to evaluate both functionalized (QD+) and non-functionalized nanoparticles. Aliquots of both QD-BRET nanoparticles were prepared for (1) *transmission electron microscope* or TEM imaging (Jeol 2100 Lab6 200 kV TEM operated at 200 kV), using a standard protocol; (2) *dynamic light scattering* (DLS; zetaPALS @ 659 nm) diameter size measurements at 37°C, after suspension in water and 5 min equilibration, and measurements (total of 5 for each nanoparticle type) done every 2 min using the NNLS algorithm for particle size; and (3) a 1% a*garose gel electrophoresis*, with samples being resolved (1X TBE buffer; 45 min at 110 V) and gels visualized and imaged on a UV light transilluminator.

### Gamete preparation and labeling

*High motile and living spermatozoa* were purified by centrifugation (600 g for 30 min) through a monolayer percoll gradient (PorciPure, Nidacon; Mölndal, Sweden). Resulting pellets of living spermatozoa, devoid of any contaminations (i.e., dead/abnormal sperm, somatic cells, and virus and bacteria if any) were suspended in the washing medium and centrifuged (250*g*, 5 min) to remove the remaining percoll. Sperm pellets were resuspended in the washing medium, counted (SpermaCue Photometer; Minitube of America, Verona, WI, USA), and concentrations adjusted to 2 × 10^8^ spermatozoa/ml of washing medium. Sperm aliquots (0.5 ml) were mixed with various concentrations of nanoparticles and incubated for 30 min at 37°C, under a gently agitation.

*Cumulus*-*Oocyte complexes* were aspirated from healthy ovarian follicles, washed and transferred in four-well dishes (Nunc; Sigma-Aldrich) containing 0.5 ml of maturation medium supplemented with various concentrations of nanoparticles. Maturation took place in an incubator set at 38.5°C under 5% CO_2_, in a humidified environment, as previously described [25]. After 1 h maturation, groups of COCs were collected from the each treatment groups and considered as immature COCs. The remaining COCs were collected after full-term maturation of 44 h.

Nanoparticles were used at concentrations of 0 nM QD− (QD0), 0.1 nM QD− (QD0.1−), 1 nM QD− (QD1−), and 1 nM QD+ (QD1+) to label both gametes. Concentrations of 0, 0.1, and 1 nM QD respectively corresponded to 0, 0.3 × 10^11^, and 3 × 10^11^ nanoparticles and all experiments were repeated four times with independent sample collections (semen or ovaries).

### Evaluation of sperm labeling and viability

Immediately after labeling, a 4 × 2 factorial arrangement of spermatozoa was used to evaluate the acrosome membrane integrity. The four labeled-groups of spermatozoa (QD0, QD0.1−, QD1−, and QD1+) were incubated with 0 or 1.5 μg/ml of FITC-PSA dye in the washing medium (Sigma-Aldrich) for 20 min at 37°C. After two washes (1,000 g—3 min) to remove the excess of dye, spermatozoa were suspended in PBS. Non-labeled spermatozoa were incubated with 0 or 10 µM Ca^2+^ ionophore A23187 (Sigma-Aldrich). The presence of ionophore served as a positive control to induce acrosome reaction. All samples were subjected to a flow cytometry analysis of the QD labeling and FITC-PSA staining. The flow cytometer (Becton–Dickinson FACSDiva version 6.1.3) was equipped with a quantum dot 655 nm filter and a 488 nm argon laser excitation. The proportions of sperm labeling in each factorial arrangement and controls (samples with or without ionophore) were evaluated. Experiments were repeated three times with independent sample collections and a minimum of 3,000 sperm cells were analyzed per sample in each experimental replicate.

### Tissue preparation and labeling

*Ovarian follicles* A protocol developed by Wu et al. was used with a minor modification in this study [26]. Briefly, healthy antral follicles (4–8 mm in diameter) were dissected from pig ovaries in North Carolina State University-23 (NCSU-23) holding medium supplemented with 3% BSA-Fraction V (mg/ml). Dissected and trimmed follicles were placed in the culture medium consisting of NCSU-23 medium supplemented with 3 mg/ml BSA, 1% (v/v) Insulin-Transferrin-Selenium (ITS), 1.5 ng/ml porcine follicle-Stimulating hormone (pFSH), 30 ng/ml human Luteinizing hormone (hLH), 7.5% (v/v) porcine serum, and 1% (v/v) Penicillin/Streptomycin. The culture took place in 24-well tissue culture plates with 2–3 follicles per well containing 2–3 ml of culture medium. After 24 h of culture at 37°C under 5% CO_2_, in a humidified environment, follicles were microinjected (FemtoJet microinjection system; Eppendorf, Hauppauge, NY, USA) with 5 µl of PBS or 60 pmol (in 5 µl) QD1− or QD1+ , and follicles were returned to culture (Day 0) for an additional 4 days (Day 4). Half of culture media was renewed every other day, from Day 0. Subset of follicles was not microinjected for later use as controls, for auto-fluorescence or -luminescence.

*Reproductive tracts* On the day of experiment, reproductive tracts of gilts were freshly collected at a local abattoir and transported to the laboratory on ice. All genital tracts were washed several times, cut into anatomic sections (uterine body, uterine horn, and oviduct), and all sections were kept in the washing medium until use for imaging. One tract was used for each replicate and a total of three independent replicates were performed.

### Bioluminescence imaging

After labeling, all groups of *spermatozoa* (10^8^/0.5 ml *of labeling medium*) were washed three times by centrifugation (1,000×*g*-3 min) to remove the excess of nanoparticles. Sperm pellets were resuspended in 50 μl of washing medium and kept into 1.5-ml Eppendorf tubes for in situ imaging. For ex vivo *intra*-*uterine imaging*, labeled (QD-) and unlabeled (QD0 or control) spermatozoa were separately loaded into 0.5-ml plastic straws that were subsequently placed outside and inside the reproductive tract sections for imaging. Groups of approximately 50 *labeled and unlabeled COCs* were manipulated separately and washed three times by successive steps, followed by their transferred into a 96-well black bottom plate containing 50 μl of PBS for imaging. *Ovarian follicles* were collected on Day 1, Day 2, and Day 4 post-nanoparticles microinjection.

For bio-imaging, sperm suspensions and COCs were respectively supplemented with 4 and 2 μg of coelenterazine, while follicles were microinjected with 750 ng. All samples were immediately imaged (within 5 min) for bioluminescence signal or photon emission using the IVIS 100 imaging system (Perkin Elmer, Hopkinton, MA, USA), as previously reported [5]. The total photon emission data were recorded as photons/s. However, the proportion of light transmitted and captured across the surface of genital tract tissue sections was calculated as the total photon emission of luminal labeled spermatozoa (Inside) divided by the total photon emission of labeled spermatozoa outside of the genital tract [(Inside/Outside) × 100].

### Fluorescence, hyperspectral, and transmission electron microscope imaging

After bioluminescence imaging, all samples were kept for in situ fluorescence imaging of quantum dots within the cells. Labeled and non-labeled samples were fixed in 4% methanol-free paraformaldehyde (spermatozoa and COCs) or 10% formalin (follicles) solutions. Fixed follicles were submitted to histological preparation, and sections of 4–6 μm thickness were generated. All other samples were smeared or placed on histology slides, rinsed, and mounted with appropriate medium for imaging.

*Laser fluorescence imaging* was performed with the confocal microscope (LSM510, Carl Zeiss Micro Imaging GmbH, Jena, Germany). For direct imaging, anti-human plasminogen antibody (Santa Cruz Biotechnology) was used for in situ immunofluorescence detection using FITC-conjugated secondary antibody as previously described [27]. After mounting on microscope histology slides with medium containing DAPI to counterstain nuclei, slides were used for confocal fluorescence imaging. Microscope filter sets of 420/40 Excitation, 660/40 Emission, and 475 DCXR dichroic were used and the background fluorescence of samples without nanoparticles served as the control.

*Optical and hyperspectral imaging* were performed with the CytoViva® imaging technology (CytoViva Inc.; Auburn, AL, USA). Spatial and spectral data were collected in each pixel of spermatozoa, COCs, and follicle sections that were fixed on histology glass slides. Hyperspectral data were quantified (CytoViva Hyperspectral Image Analysis Software algorithm). PBS-dispersed quantum dot nanoparticles were used to create the reference spectral library by comparing the particle filter results to a negative control sample, which removes any false positive spectral data and ensures valid results. Dark-field optical images of samples were taken and scanned with the reference spectral library to match pixels corresponding to the nanoparticles. Matching pixels were mapped in a pseudo red color to illustrate the presence and location of the nanoparticles in cells.

*Transmission electron microscopy* was performed on spermatozoa only. Sperm samples previously labeled with functionalized (QD+) or non-functionalized (QD−) QD-BRET were prepared for TEM as previously described [5]. Images were taken with the Jeol 2100 Lab6 200 kV TEM, operated at 200 kV.

### Statistical analyses

Analyses were performed with the IBM SPSS 22.0 software package. One way-ANOVA was used to test the effect of the nanoparticle labeling (fixed factor) on all dependent factors (bioluminescence and fluorescence intensities and viability data). When statistical differences were observed p < 0.05), analyses were followed by pairwise comparisons (Fisher’s LSD post hoc test). All data are expressed as mean ± SEM, unless otherwise indicated.

## Results and discussion

Numerous challenges associated with deep-tissue characteristics drastically limit the broad application of bio-imaging technologies in large animals [28]. Especially in the reproduction field, the need of high spatio-temporal resolution and satisfactory contrast imaging technologies to detect spermatozoa and perform molecular or functional analyses are essential to overcome these challenges [1, 29]. Here we tested the interaction of QD-BRET (QD−) nanoparticles with pig gametes and ovarian follicles, for non-targeted and targeted bio-imaging in in vitro (labeled-spermatozoa and -oocytes), ex vivo (intra-uterine labeled-spermatozoa), and in situ (labeled-follicles during culture) settings. Previous studies have shown the non-toxicity of the QD− nanoparticles on boar spermatozoa [5] and other somatic cells [14, 16, 20, 30], when used at appropriate concentrations. For targeted imaging, we used QD− nanoparticles that were tagged with anti-porcine plasminogen antibody (QD+).

Plasminogen is an inactive zymogen mainly synthesized by the liver and at lesser extent in other tissues such as testes [31, 32]. Its secretions are found in various extracellular fluids, including seminal plasma and oviductal fluids [33, 34]. Plasminogen can specifically bind to both gametes and its conversion into the serine protease plasmin by sperm-bound urokinase-type plasminogen activators (u-PAs) contribute to the regulation of fertilization [22, 23, 34, 35].

### Evaluation of synthesized QD-BRET nanoparticles

**Figure 1:**
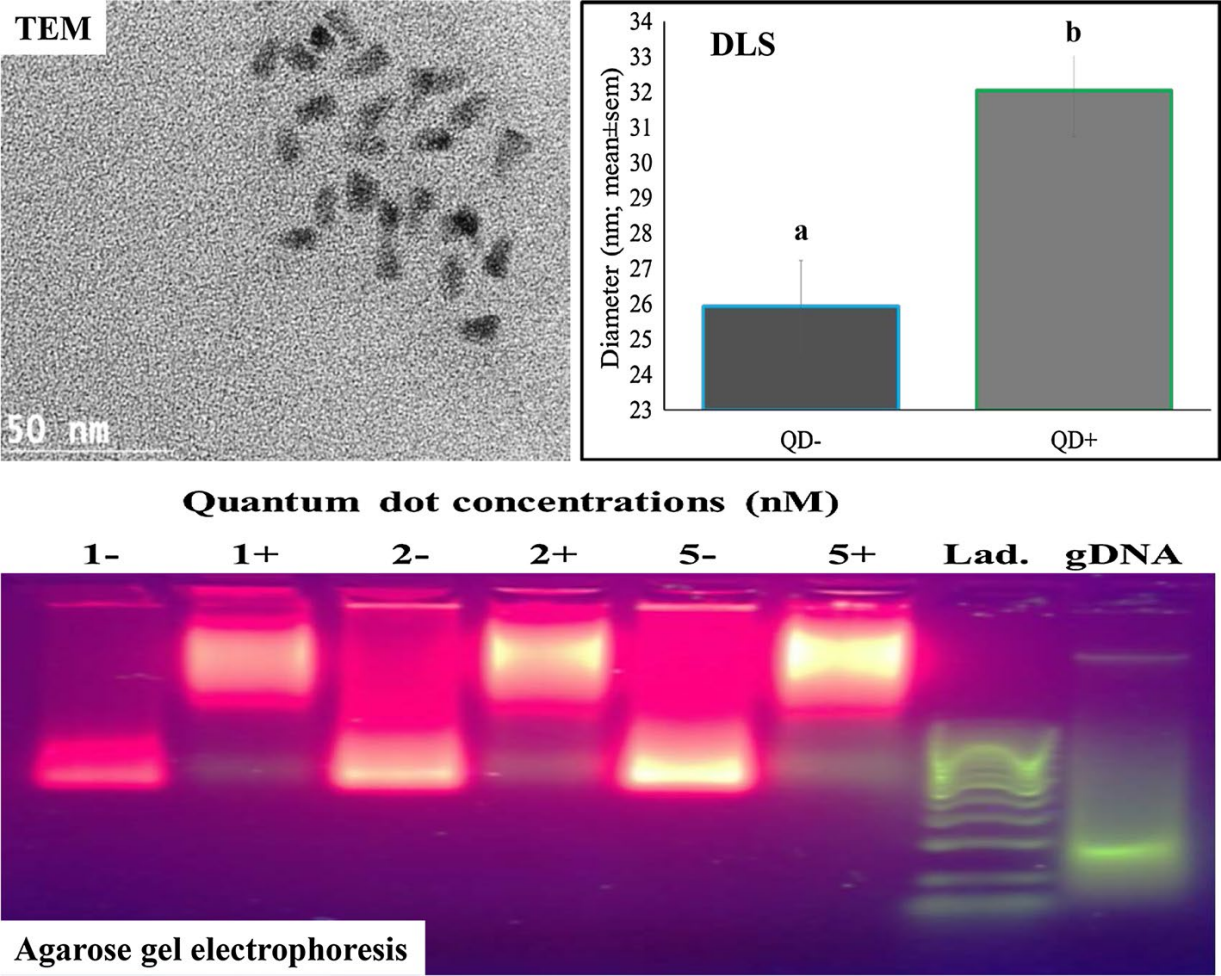
Characterization of the designed QD-BRET nanoparticles. Nanoparticles were analyzed with transmission electron microscopy (TEM), dynamic light scattering (DLS), and agarose gel electrophoresis. The TEM image shows the core–shell of the quantum dot nanocrystals averaging 8.2 ± 1.7 (SD, of 20 measurements); while the DLS measured the diameter sizes (n = 5) of the functionalized (32 ± 1.3 nm) vs. non-functionalized (26 ± 1.3 nm) QD-BRET that were significantly different (*a*, *b*; P < 0.05; T test). The agarose gel shows differential migration of both nanoparticles, with functionalized aliquots (1+ , 2+ , and 5+) being heavier and migrating slowly than their non-functionalized counterparts (1−, 2−, and 5−). The gel was loaded with various concentrations of nanoparticles (1, 2, and 5 nM/well, equivalent to 3 × 10^11^, 6 × 10^11^, and 15 × 10^11^ nanoparticles, respectively). A 100 base pair (bp) PCR DNA ladder (Lad.) and sperm genomic DNA (gDNA) were loaded alongside for quality control of the gel electrophoresis.

All data are summarized in Figure 1. The calculated average (±SD) of random measurements of QD core–shell (CdSe/ZnS) diameters through the transmission electron microscopy (TEM) images was 8.2 ± 1.7 nm, which appeared little bit higher than our previous report [5] using a different batch of QD-BRET nanoparticles prepared by the same company (Zymera, Inc.). The tediousness of measuring quantum dot core–shells on the TEM may likely explain variations observed between studies. Both QD− and QD+ stocks were dispersed in the PBS (pH 7.4) solution used to label spermatozoa, for hydrodynamic dynamic light scattering size measurements. Analyses clearly showed functionalized (QD+) and non-functionalized nanoparticles with significantly different diameter sizes by intensity (32 ± 1.3 vs. 26 ± 1.3 nm, respectively; P < 0.05—*t* test) or by volume (40.4 ± 1.8 vs. 32.2 ± 0.9 nm, respectively; P < 0.05—t test). Both solutions showed presence of aggregates that appeared larger in QD− (239 ± 8.6 nm) than QD+ (153 ± 3.7 nm) samples (Additional file 1: Figure S1). The use of undispersed samples straight from the stocks to agarose gel electrophoreses confirmed the size difference of both nanoparticles. The functionalized nanoparticles (QD+) were heavier and slower to migrate through the gel than their non-functionalized counterparts (Figure 1), although we cannot tell whether the gel imaging is showing aggregate or non-aggregate sizes. The technical approaches used and results obtained with non-functionalized nanoparticles are consistent with a previous study verifying the conjugation of luciferase (Luc8) to quantum dot emitting at 655 nm [6].

### Gamete labeling and bioluminescence imaging

**Figure 2:**
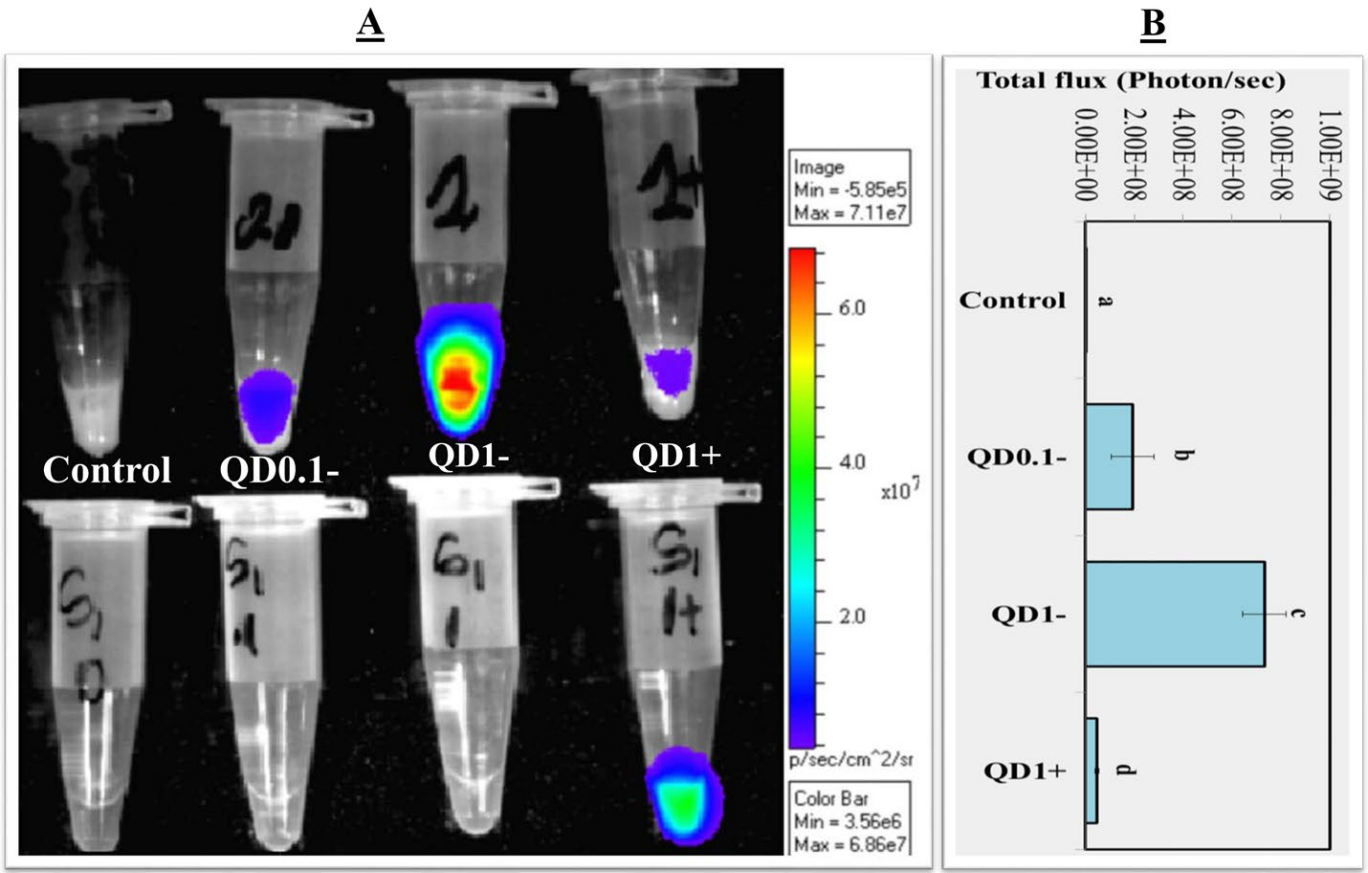
Bioluminescence imaging (BLI) of spermatozoa. **A** BLI of spermatozoa labeled with 0 nM (Control), 0.1 nM (QD0.1−), and 1 nM (QD1−) QD-BRET. Spermatozoa labeled with QD conjugated with anti-plasminogen antibody (QD1+) are also shown. Corresponding supernatants containing excess of QD− or QD+ are also imaged (*bottom tubes*, hand-marked S1). All samples were mixed with coelenterazine, the luciferase substrate before BLI. The quantification of total signals (photons/s) is summarized in **B**. Data are mean ± SEM of four independent replicates, and *different letters* indicate significant difference between columns (P < 0.05; ANOVA 1).

**Figure 3:**
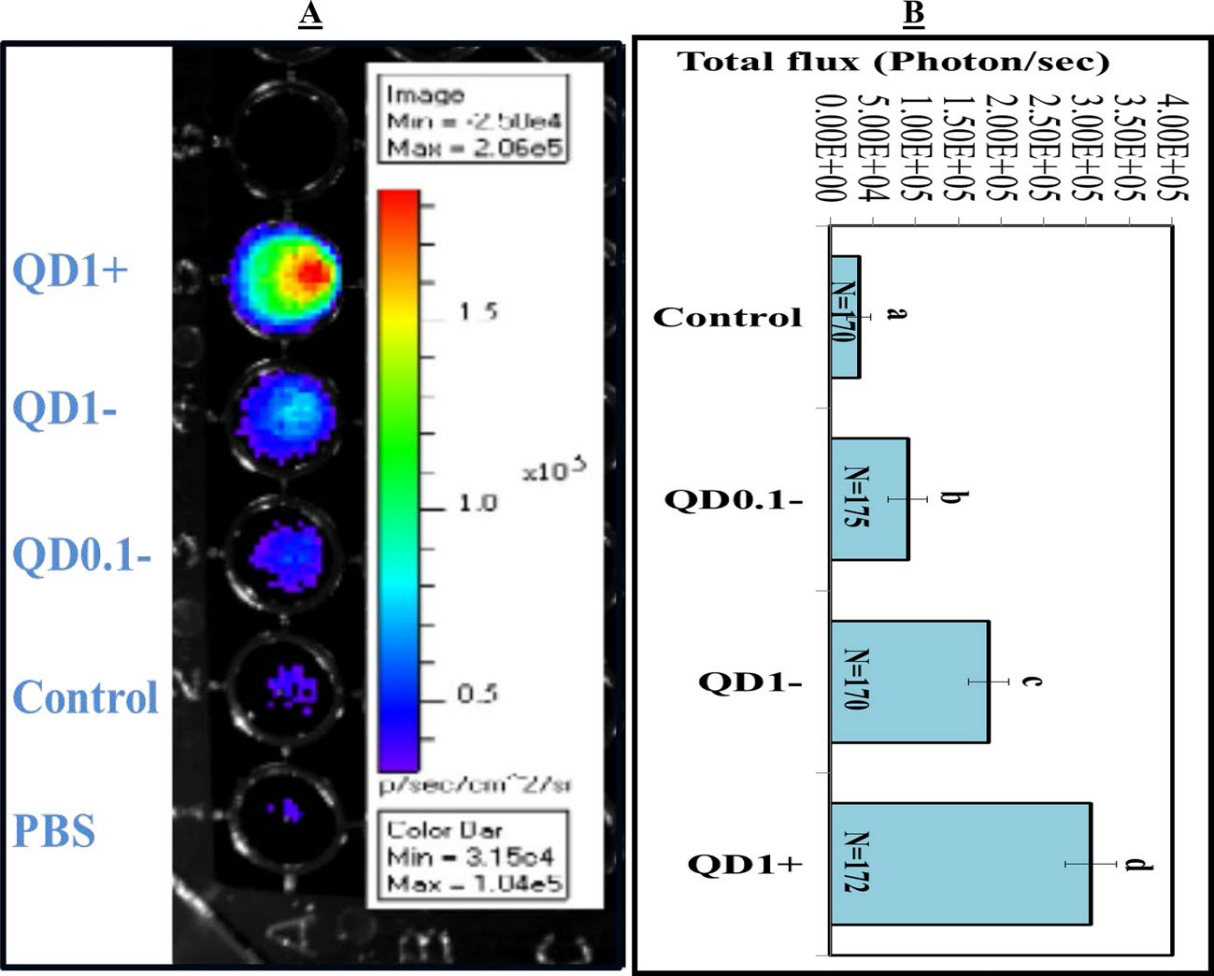
Bioluminescence imaging (BLI) of cumulus oocyte complexes (COCs). **A** BLI of COCs matured in the presence of 0 nM (Control), 0.1 nM (QD0.1−), and 1 nM (QD1−) QD-BRET and 1 nM QD-BRET conjugated with anti-plasminogen antibody (QD1+). All samples were mixed with coelenterazine, the luciferase substrate, before BLI. The quantification of total signals (photons/s) is summarized in **B**. Imaging of each experimental replicate a included well without COCs, but filled with comparable volume of PBS as in the experimental groups to allow the evaluation of the PBS autofluorescence contribution to the results. Data are mean ± SEM of four independent replicates. Columns with *different letters* are significantly different (P < 0.05; ANOVA 1).

Spermatozoa incubated and cumulus-oocyte complexes (COCs) matured in the presence of 0, 0.1, and 1 nM QD− showed dose-dependent like production of bioluminescence (light) emission (Figures 2, 3). In comparison to their respective controls, the total fluxes (photons/s) were increased by 59× and 223×, in spermatozoa (Figure 2) and 1.6× and 1.7×, in COCs (Figure 3) by the presence of 0.1 and 1 nM QD− (P < 10^−4^; ANOVA 1). In contrast, lower and higher bioluminescence signals were respectively observed in spermatozoa and COCs labeled with 1 nM QD+ than their counterparts with 1 nM QD− (P < 0.05; ANOVA 1).

Data with spermatozoa are in agreement with our previous report [5], describing interaction of QD-BRET (QD−) nanoparticles with boar spermatozoa without affecting their function (motility and fertilization). Here we report for the first time the incorporation of QD− by porcine COCs, while their interactions with functionalized nanoparticles (QD+) indicate the presence of plasminogen protein in both gametes. This successful interaction were further confirmed through transmission electron (Additional file 2: Figure S2) and confocal laser (Additional file 3: Figure S3) microscopy analyses, showing higher accumulations of quantum dots in spermatozoa (TEM) that corresponded to similar area targeted by anti-plasminogen antibody (the sperm head). Overall and despite using different sources of antibodies (Acris and Santa Cruz), generated images appear alike, which position the use of nanoparticle-based imaging as a viable approach for non-invasive functional analyses of desired proteins, such as the plasminogen/plasmin system, having putative role during fertilization [22, 24]. The current direct and indirect (through nanoparticles) findings bring further confirmation of the presence of plasminogen on mammalian oocytes [23], while revealing its presence in spermatozoa that has not yet been clearly demonstrated in the literature.

### Ex vivo intra-uterine bioluminescence imaging of spermatozoa

Accurate and non-invasive investigations of mammalian gametes in physiological conditions are limited with the conventional techniques and imaging approaches [36–39]. Consequently, the progression and behavior of gametes, especially spermatozoa within the female reproductive tract remains a mystery. As a first step toward to elucidating this physiological event, the current experiment was developed for a possible tracking of spermatozoa through the capture of luminal sperm-emitting photons over the reproductive tract surface.

**Figure 4:**
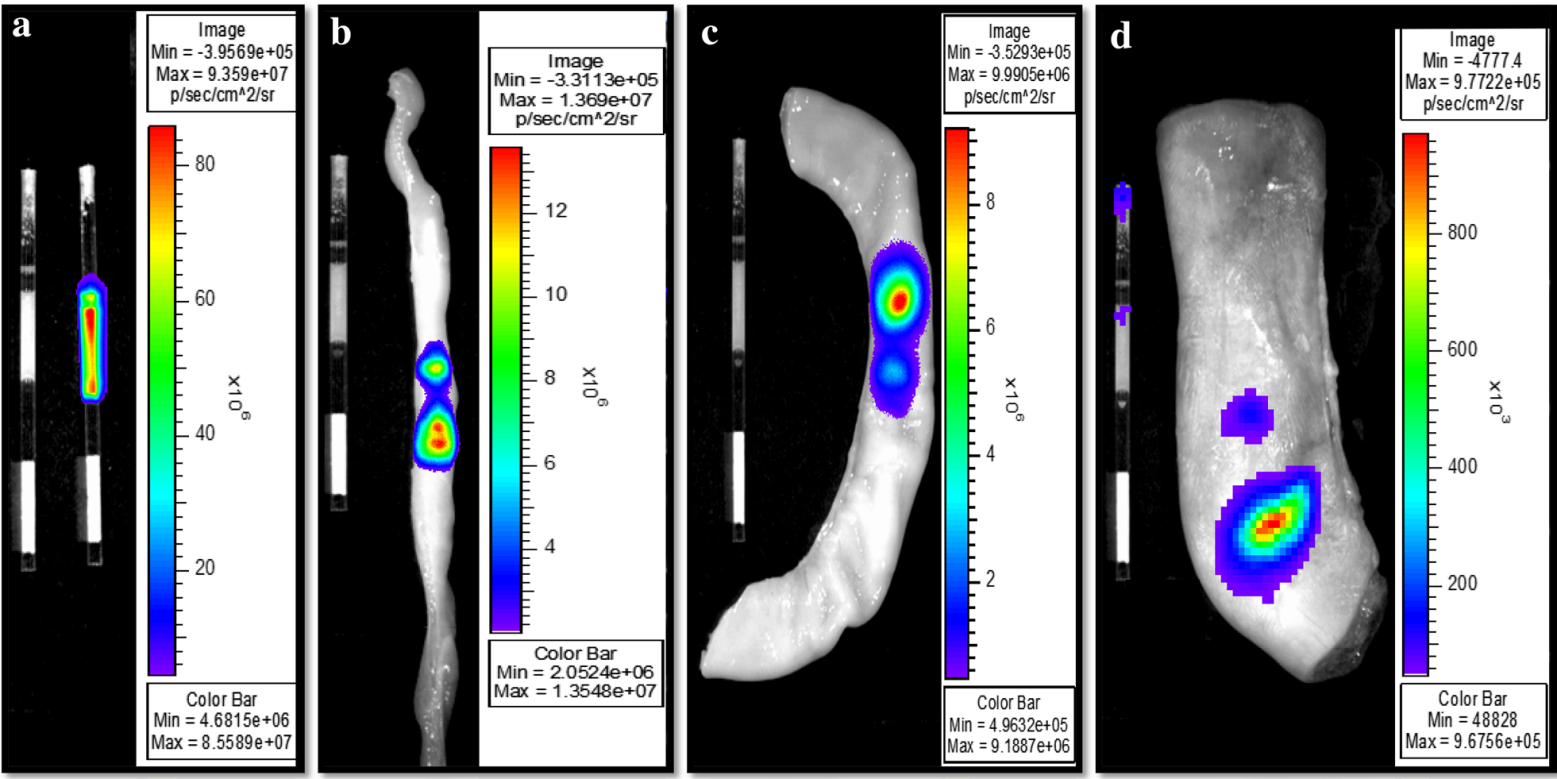
Representative intra-uterine bioluminescence imaging of labeled spermatozoa. Spermatozoa were labeled or not with 1nM QD-BRET (QD1−), loaded into 0.5-ml plastic straws and imaged inside and outside each reproductive tract sections. Bioluminescence emissions were captured ex vivo, outside (**a**) and on the surface of oviduct (**b**), uterine horn (**c**), and uterine body (**d**) sections. The ratios of outside over inside luminescence signals (×100) were used to express and interpret data between sections.

Bioluminescence signals (photons/s) were captured above background on each reproductive tract section (Figure 4). A higher proportion of light was transmitted through the oviduct section (21.5 ± 2.4%), compared to the uterine horn (7.2 ± 0.9%) and body (1.1 ± 0.4%) (P < 0.01; ANOVA 1). The low proportions of transmitted lights underline the challenge of deep-tissue imaging, while indicating the possibility of using nanotechnology approach for successful imaging of spermatozoa within the genital tract lumen. The current imaging was based upon the bioluminescence property of the QD-BRET, which brightness is till limited for deep tissue imaging. Yet, the red fluorescence capability of these nanoparticles remains to be exploited.

The inorganic nature of quantum dots (QD) offers a great potential for bright and photo-stable fluorescence signal to allow intra-uterine imaging of spermatozoa. However, the successful utilization of QD nanoparticles for the purposed-bio-imaging may fully rely on miniaturized devices, such as the probe-based confocal laser endomicroscope (pCLE). This instrument uses light in the visible spectrum that should be enough to excite the QD nanoparticle, and provide adequate spatial resolution by the mean of flexible miniaturized fiber optic probes of 1.5–2.6 mm outer diameter. The pCLE is being used for imaging diagnoses in the clinical area [40–42], and has been tested for ex vivo studies of spermatozoa in human testicle [3] and ewe oviduct [43] using organic fluorochromes.

### Sperm labeling and viability evaluation through flow cytometry

**Figure 5:**
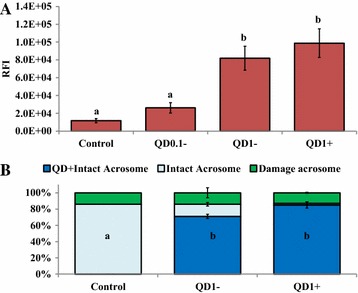
Flow cytometry evaluations of sperm labeling and viability. Spermatozoa were labeled with 0 nM (Control), 0.1 nM (QD0.1−), and 1 nM (QD1− and QD1+) nanoparticles, followed by their incubation with FITC-PSA to the acrosome integrity or intactness. Proportions of 0%, 76 ± 4%, and 91 ± 2% spermatozoa were labeled with nanoparticles in the Control, QD0.1, QD1−, and QD1+ groups. The mean fluorescence intensities of nanoparticles-labeled spermatozoa (**A**) and proportions of spermatozoa with intact and damage acrosomes (**B**) were evaluated. Spermatozoa incubated with 0 and 10 µM calcium ionophore served as negative and positive controls, respectively. Columns (RFI, in **A** and QD+ intact acrosome, in **B**) with *different letters* differ significantly (ANOVA-1; p < 0.05). Data are mean ± SEM of four independent replicates.

Flow cytometry is an extensively used approach to study a variety of single sperm attributes with high precision, accuracy, and low costs using fluorescence techniques [44]. Proportions of 64 ± 4.5%, 76 ± 4%, and 91 ± 2% of spermatozoa were successfully labeled with 0.1 nM (QD0.1−), 1.0 nM (QD1−), and QD1+ compared to nothing in the control or QD0 group (P < 0.05). Surprisingly, the mean relative fluorescence intensities (RFI) of QD1− and QD1+ remained comparable, but were both significantly higher than those in the control and QD0.1− (Figure 5A; P > 0.05). We attributed this discrepancy to the well-known brightness fluorescence of quantum dots, while the co-functionalization of QD-BRET with luciferase (Luc8) and antibody may limit the amount of Luc8 per QD, leading to low bioluminescence emission. Nevertheless, the findings suggest that most spermatozoa express plasminogen protein, and the limited-expression on the sperm surface, mostly in the head area, may result in a low overall bioluminescence emission.

As for the viability of labeled spermatozoa, the intactness of their acrosome membrane was evaluated. The control group showed a proportion of 86% of spermatozoa with intact acrosome, while 85% of spermatozoa labeled with QD1+ versus 71% with QD1− maintained intact acrosome membrane (Figure 5B; P < 0.05). This finding indicates the possibility of small-sized QD-BRET (20–25 nm) to damage the sperm acrosome membrane. In possible support of this speculation, a recent study has reported the internalization of small-sized gold nanoparticles (less than 10 nm) and the exclusive localization of larger nanoparticles on the plasma membranes of intact bovine spermatozoa [45]. In the other hand, the absence or reduced negative effects in the QD1+ group may be attributable to the bigger size of functionalized nanoparticles (32 vs. 26 nm to QD1−) and more likely to their specific interactions with plasminogen proteins found on the sperm surface membrane. Furthermore, the formation of nanoparticle aggregates are another potential sources of toxicity to cells, including spermatozoa. Barchanski et al. have detected higher accumulations of conjugated-gold nanoparticles in the post-equatorial region of acrosome-reacted spermatozoa, which commend for further and close investigations of quantum dot interactions with spermatozoa [45]. These investigations will contribute to further optimize the use of quantum dots for non-invasive imaging of fully functional and viable spermatozoa. Great interests are expected from controlled-uses of nanoparticles for sperm labeling in assisted reproduction. Here we used a total of 3 × 10^11^ nanoparticles to label 100 million spermatozoa, corresponding to 3,000 nanoparticles per sperm cell that is well-below the 14,000 gold nanoparticles inducing detrimental effects on bovine spermatozoa [46]. The current findings complement a previous report indicating no major losses of motility, plasma and mitochondrial membrane integrities, and fertilizing potential of pig spermatozoa labeled with 1 nM QD (or QD1−), equivalent to 3,000 nanoparticles/sperm cell [5].

### Ovarian follicle labeling and bioluminescence imaging

The ovarian follicle growth is concomitant with the oocyte development and the formation of an intra-follicular cavity surrounded with various cell types. This cavity is filled with a fluid resulting from blood exudates and other follicular cell secretions, which dynamic concentrations in molecules such as hormones and growth factors regulate the growth of both follicle and oocyte [47, 48].

**Figure 6:**
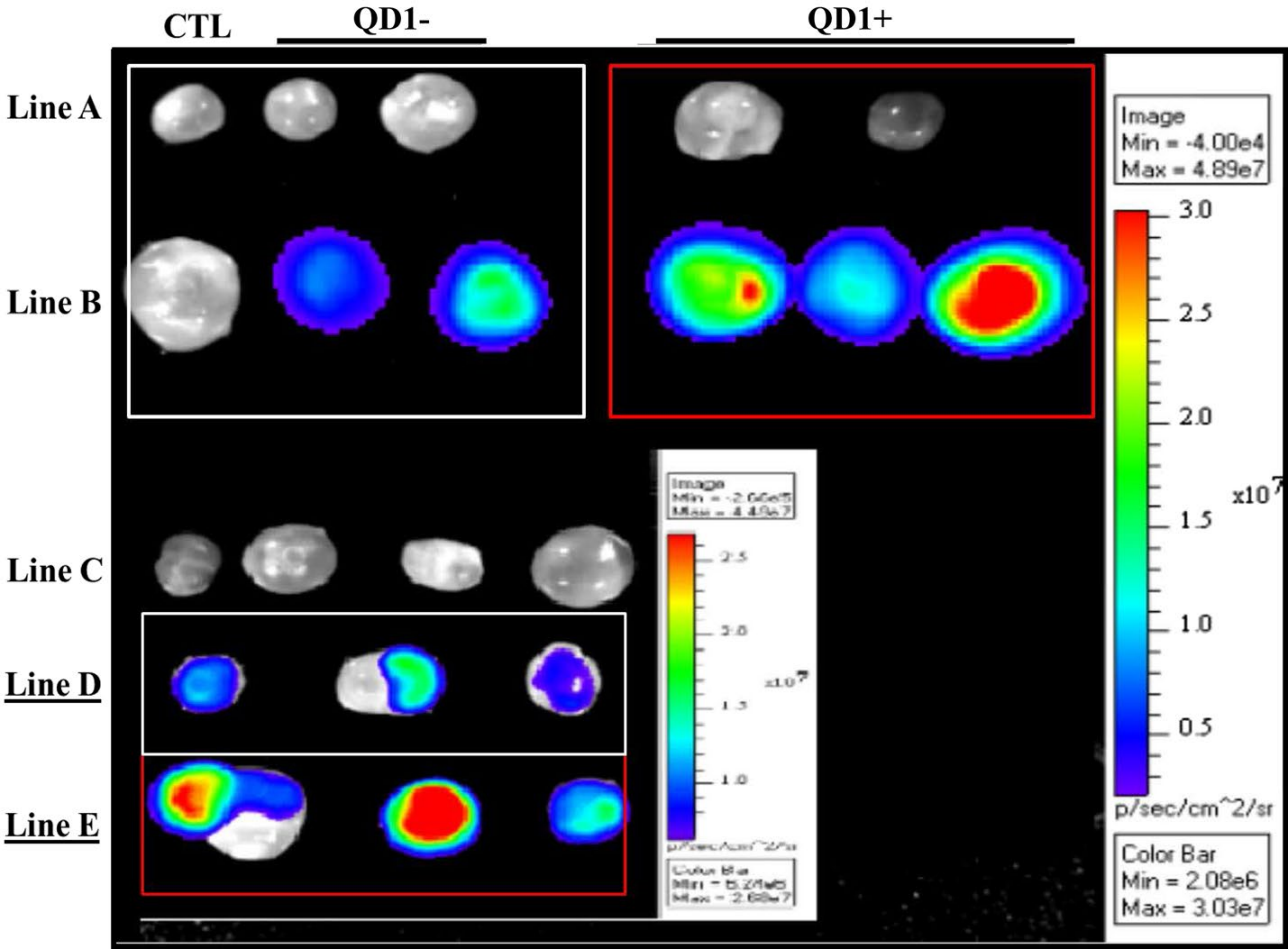
Bioluminescence imaging of QD-BRET (QD) microinjected cultured ovarian follicles. Follicles were microinjected with QD1− or QD1+ during in vitro culture. Imaging was performed with follicle controls, microinjected with only PBS (*Line A*, CTL), QD1− (*Line A*, *White Box*), or QD1+ (*Line A*, *Red Box*), follicles microinjected with both coelenterazine and QD1− (*Line B*, *White Box*), or QD1+ (*Line B*, *Red Box*). The *inset* shows follicles microinjected with coelenterazine only (*Line C*) or with QD1− and QD1+ , showing respective bioluminescence emissions in *Lines D* and *E*.

**Figure 7:**
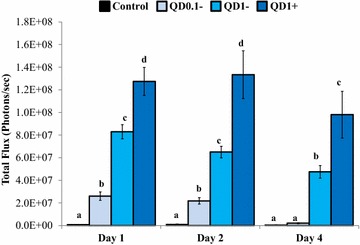
Quantification of ex vivo bioluminescence emission of ovarian follicles. Totals of 52, 28, and 32 follicles were respectively analyzed on Day 1, Day 2, and Day 4, which corresponded to 13, 7, and 8 follicles in each labeling group (Control, QD1−, and QD1+). The day of QD-BRET microinjection was considered as Day 0. Data are mean (± SEM) of three independent replicates and statistical comparisons were done within days (Day 1, Day2, or Day 4). Columns with *different letters* indicate significant differences (P < 0.05; ANOVA 1).

Here, the microinjection of QD nanoparticles within the follicular cavity constituted an attempt to develop a strategy for intra-follicular imaging for biosensing and tracking of key molecules that intra-follicularly influences oocyte quality and viability. Figure 6 shows bioluminescence imaging of QD-BRET nanoparticles with (QD1+) or without (QD1−) the plasminogen antibody within the ovarian follicles. Follicles microinjected with QD1+ always displayed greater luminescent signals than those with QD1− (Figure 6; Line B in Red box vs. White box and Line E vs. Line D). No signals were observed in the control groups, consisting of follicles microinjected with either PBS (CTL, Line A), coelenterazine (CTL, Line B), or QD1− without coelenterazine (Line C). Interestingly, the microinjection of QD1− or QD1+ did not cross-contaminate the neighboring follicle (Figure 6; Lines D, E). This observation is important as ovarian follicles are independent structures with different health and developmental status, leading to the production of oocytes with different developmental competences. Follicles microinjected with QD0.1− or QD1− exhibited dose-dependent like light emission at all time-points (Day 1, Day 2, and Day 4—P < 0.05; Figure 7). Interestingly, follicles microinjected with QD1 + maintained the highest light emission throughout the culture period (P < 0.05; ANOVA-1).

Although the viability of cells was not verified, data suggest that self-illuminating luminescent nanoparticles can be used for molecular imaging of cultured follicles. Our finding adds to a recent study reporting a successful intra-follicular imaging through a transgene carrying the luciferase reporter gene [49]. The current work paves the way for further optimization of this novel non-invasive imaging approach that will lead to promising in situ real-time description of the oocyte maturation and related intra-follicular key molecules.

### Confocal fluorescence microscope imaging of the nanoparticle bio-distribution

**Figure 8:**
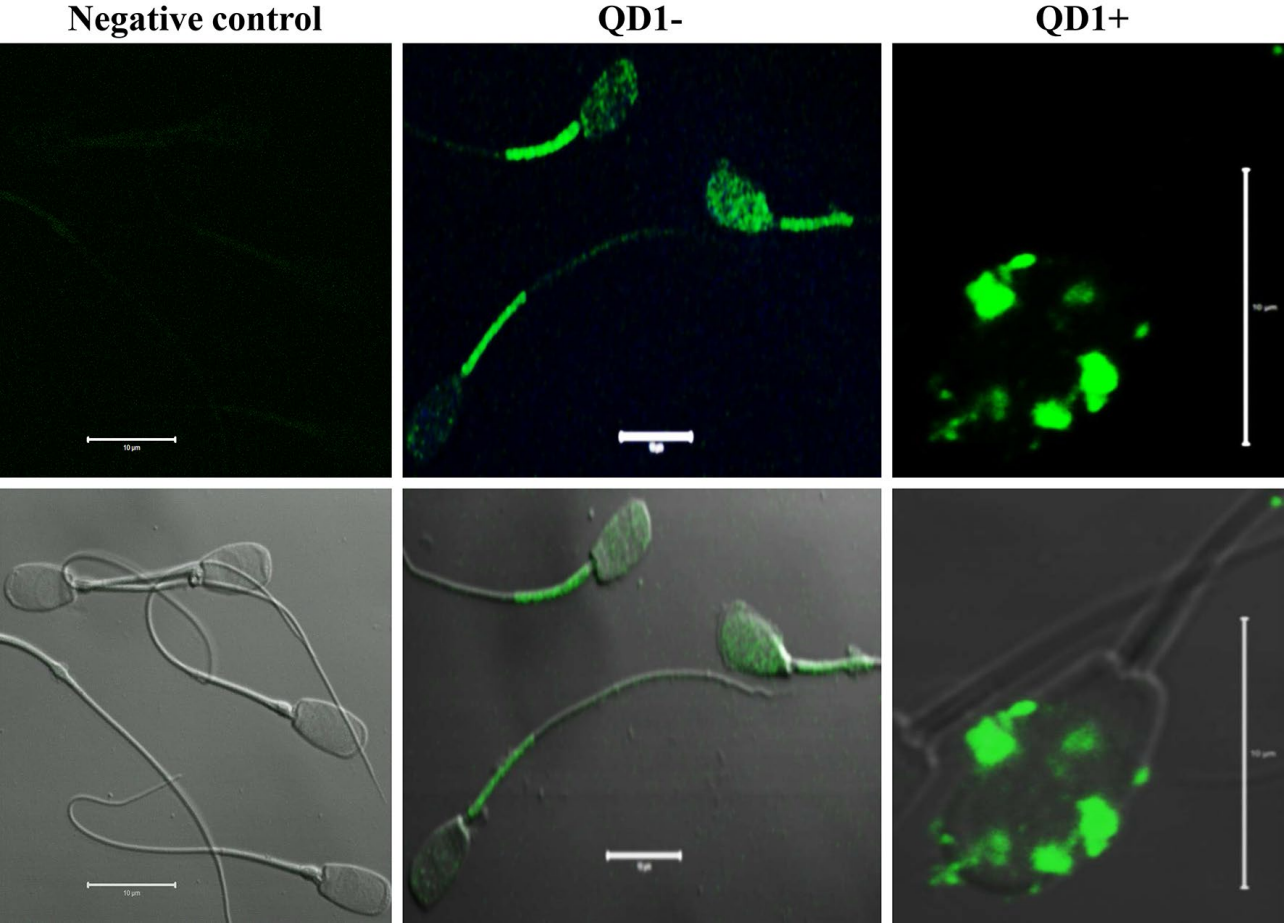
Confocal fluorescence imaging of QD-BRET labeled spermatozoa. Spermatozoa were labeled with 0 nM (control) and 1 nM plasminogen-conjugated (QD1+) or non-conjugated (QD1−) QD-BRET nanoparticles. The control group was used for fluorescence imaging settings and was designated as the negative control group. These settings were used to capture the fluorescence emission (*upper panel*). Both fluorescence and visible light signals are overlaid in the bottom panel. *Scale bar* 10 µm.

Following incubation with QD1−, the fluorescence signal appeared more diffuse on the entire spermatozoon, with the strongest signal being observed in the mid-piece section (Figure 8). This cellular distribution contrasted with the QD1+ labeling that was mainly limited to the sperm head (Figure 8). This observation brings a direct evidence of the plasminogen protein presence in boar spermatozoa. The spatial distribution of QD1− and QD1+ fluorescence signals may contribute to explain the differential bioluminescence emission between both groups.

**Figure 9:**
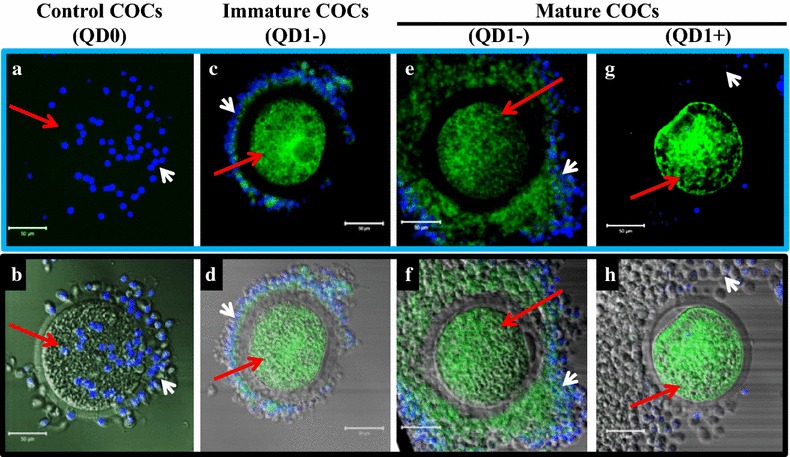
Confocal fluorescence imaging of QD-BRET (QD)-labeled cumulus-oocyte complexes (COCs). COCs were labeled with 0 nM (QD0) or with 1 nM plasminogen-conjugated (QD1+) or non-conjugated (QD1−) QD-BRET nanoparticles. Non-labeled mature or immature COCs were used to set up the imaging conditions (**a**, **b**). Micrographs in the *upper panel* (*Blue frame*) show QD-BRET fluorescence signals detected in COCs labeled before (Immature; **c**, **d**) and after (Mature; **e**, **f**, **g**, **h**) in vitro maturation. The *lower panel* (*Black frame*) shows corresponding overlaid visible and fluorescence light images. Nuclei are counterstained in *blue* with DAPI. The *white* and *red arrows* indicate the cumulus cells and oocytes, respectively.

On the other hand, the QD1− fluorescence signal appeared homogenously distributed within the oocyte and cumulus cells, before (Figure 9c, d) and after maturation (Figure 9e, f). However, this signal appeared more organized in oocytes matured in presence of QD1+, with lesser or no signal being detected in the cumulus cells (Figure 9g, h). Interestingly, the invasive detection through antibody revealed a strong accumulation of plasminogen in mature versus immature oocytes (Additional file 3: Figure S3). Both nanoparticle-based (non-invasive) and direct (invasive) detection of plasminogen showed comparable distribution within the matured oocyte.

**Figure 10:**
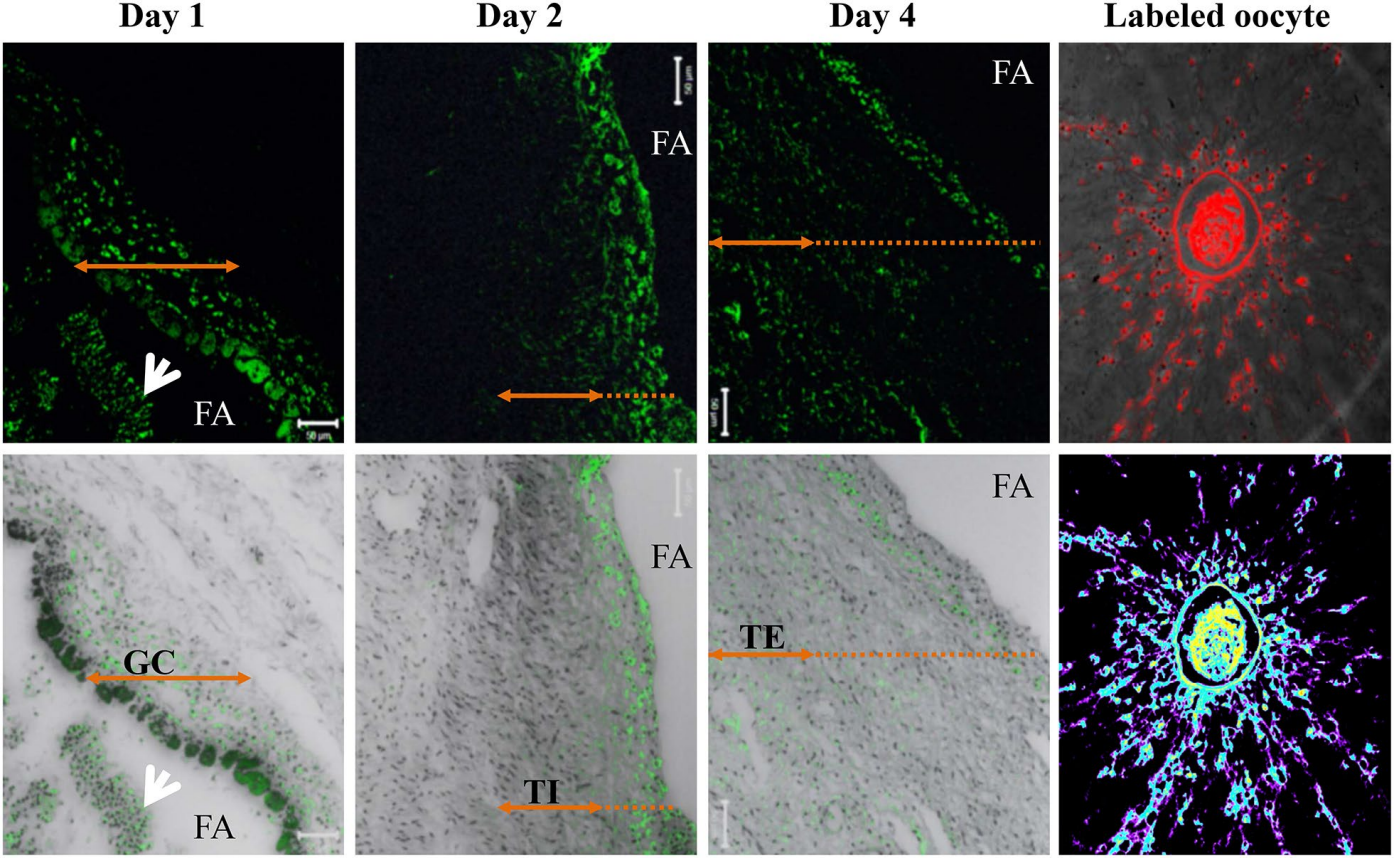
Fluorescence imaging of follicular micro-sections following QD-BRET (QD) labeling. The *upper panel* shows the intracellular progression of the fluorescence signals of QD1− nanoparticles during culture (Day 1, 2, and 4). Fluorescence signals were mainly detected in the mural granulosa cells, surrounding the follicular cavity, on Day 1 post microinjection. The fluorescence signal progressed into the mid-section of the follicle wall (theca internal cells) on Day 2, and then the entire follicle wall, including the theca external cells on Day 4. The *bottom panel* represents the corresponding overlays fluorescence and visible lights. The intra-follicular COCs also incorporated the QD1− (200×). *Arrows* indicate the extent of tissue layers (*GC* granulosa cells, *TI* theca internal, *TE* theca external). The *white arrows* show detached and stained granulosa cells within the follicular antrum (FA).

The bio-distribution of QD1− within the follicle appeared dynamic during culture. A great accumulation of the QD fluorescence signal was seen on the mural granulosa cells that surround the follicular cavity on Day 1 post microinjection. The signal appeared within the theca internal on Day 2 and then reaches the farthest layer of cells, the theca external, on Day 4. Interestingly, microinjected QD were able to penetrate the cumulus-oocyte complex, with a higher accumulation found in the oocyte (Figure 10).

Overall, the fluorescence imaging brings further confirmation of the bioluminescence observations and provides direct evidence of the plasminogen protein presence in pig gametes and ovarian follicles. Thus, nanotechnology approach based on self-illuminating quantum dots could be used as a novel methodology to measure the dynamic changes in molecular events occurring during intra-follicular growth and maturation of the oocyte. In this study, it is likely that the combination of QD-BRET ± antibody complexes with the cell-penetrating peptide, nona arginine R9, facilitates their incorporation into follicular and cumulus cells, which likely transfer incorporated-nanoparticles to the oocyte through gap-junctions uniting both cell types (cumulus cells and oocytes). The conjugation of nanoparticles with cell penetrating-peptides (CPP) has been shown effective for interactions with cells, including mammalian gametes despite different depth of penetration efficiencies between CPP [45]. Our findings using a polycationic CPP coupled to quantum dots are in agreement with a recent work showing accumulation of BSA-coated gold nanoparticles in porcine oocytes and surrounding cumulus cells [50]. These authors found no detrimental effects of gold nanoparticles on pig oocytes and spermatozoa, which is in line with our present and prior findings using quantum dots [5]. It is important to mention that these results were obtained with comparable nanoparticle diameter sizes (20 vs. 26 ± 1.3 nm for gold and quantum dot nanoparticles, respectively) and numbers (1.24 × 10^10^/COC and 1.23 × 10^3^/sperm cell for gold vs. 3 × 10^11^ and 3 × 10^3^ for quantum dot nanoparticles, respectively). Yet, numerous studies have evidenced that the penetration of nanoparticles into spermatozoa remains limited or prevented by the composition and status of the plasma and acrosome membranes [5, 46, 51].

### Hyperspectral fluorescence imaging of the nanoparticle bio-distribution

**Figure 11:**
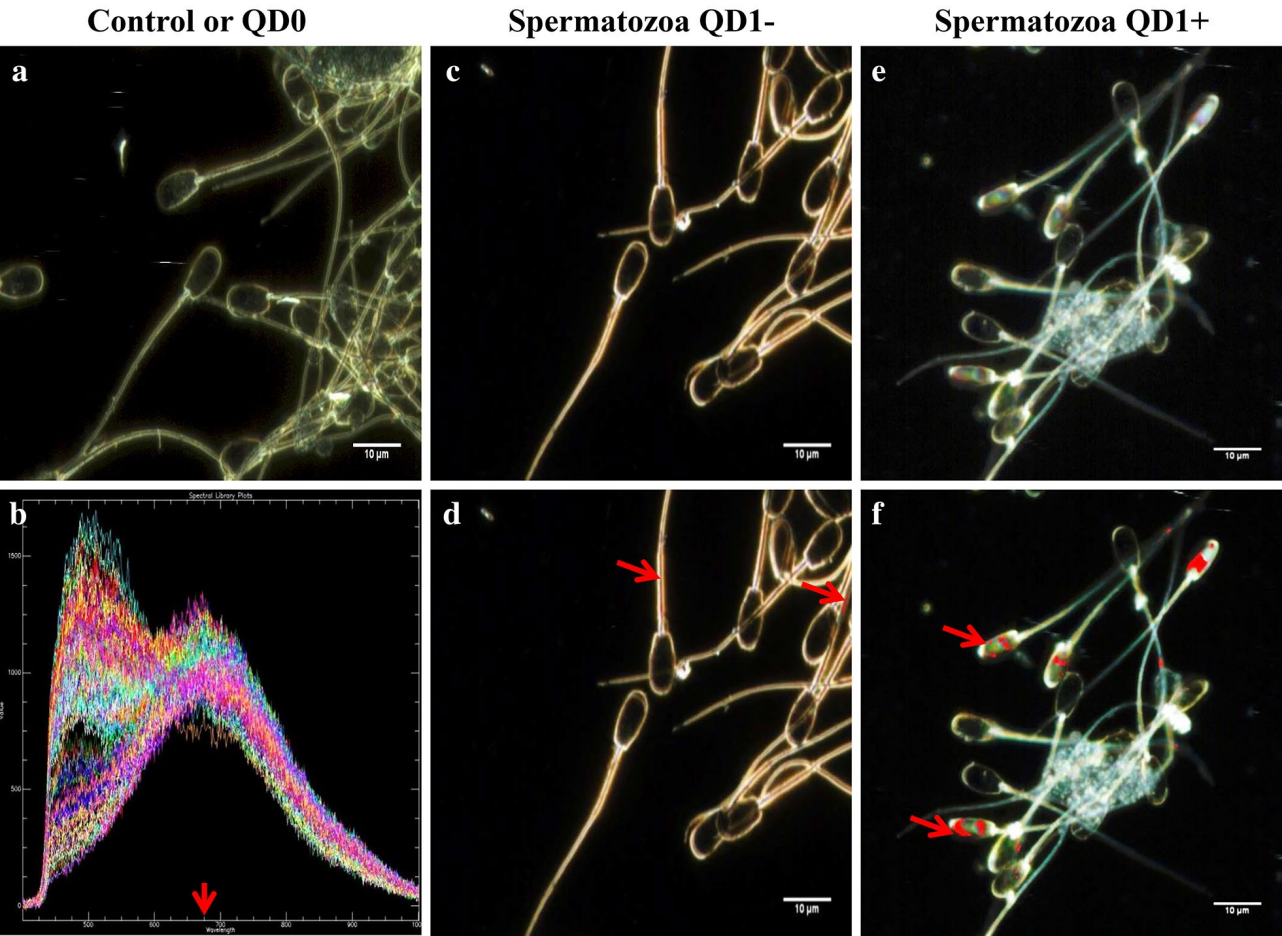
Hyperspectral fluorescence imaging of QD-BRET (QD) labeled spermatozoa. Negative control spermatozoa (**a**) were used to remove false positive signals and validation of QD-BRET fluorescence spectral libraries that were captured around or near 655 nm emission wavelength, as indicated by the *arrow* (**b**). Hyperspectral images of spermatozoa were taken (**a**, **c**, **e**) followed by the comparison of all pixels with the QD1− and QD1+ spectral libraries (Not shown here). Pixels that matched the spectral are mapped with a pseudo-red color (*arrows*) illustrating the presence and location of QD1− (**d**) or QD1+ (**f**).

**Figure 12:**
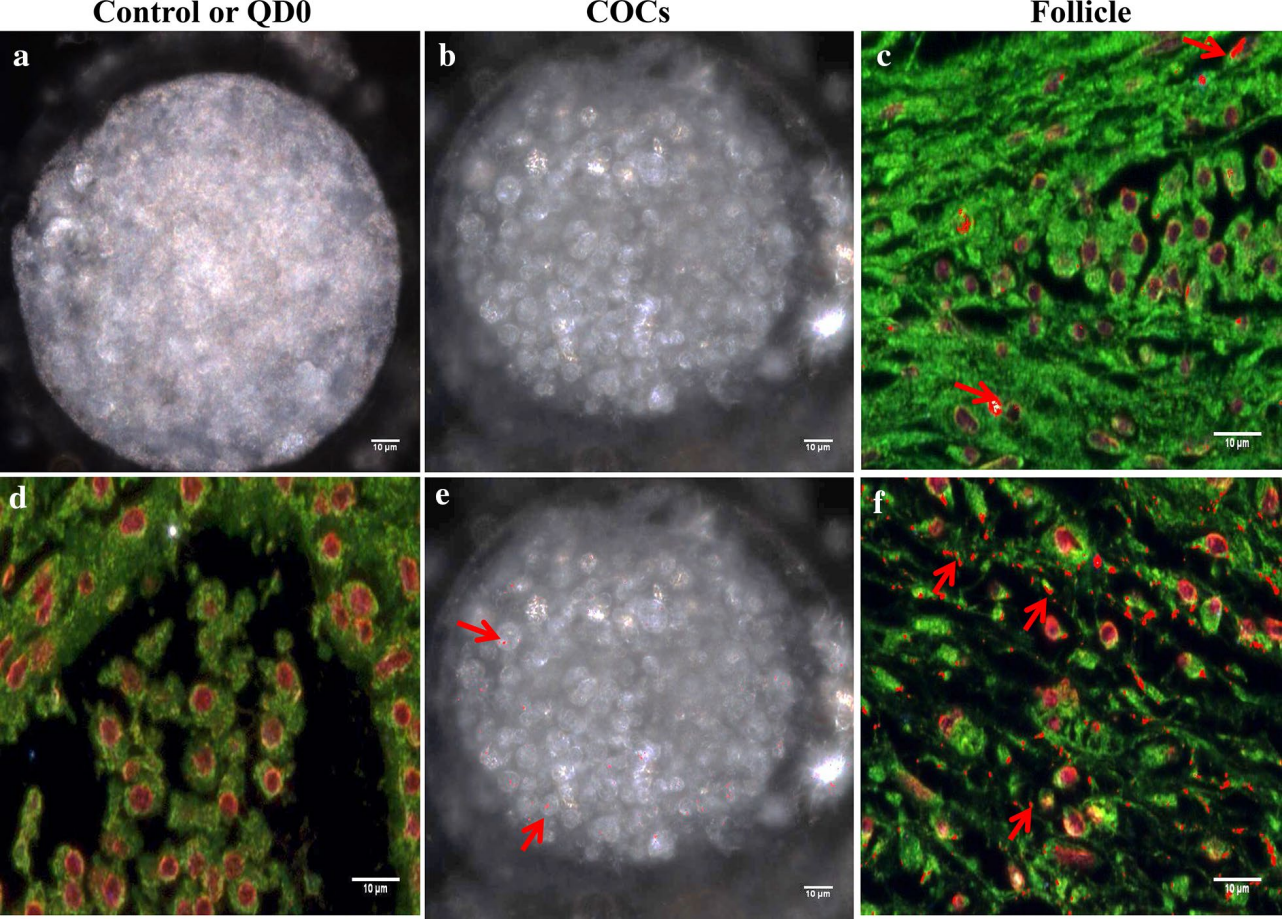
Hyperspectral fluorescence imaging of QD-BRET (QD)-labeled COCs and ovarian follicles. Micrographs show samples that were labeled with QD1− (**b**, **e**) and QD1+ (**c**, **f**). Control samples (**a**, **d**) were used to remove false positive signals and validate spectral libraries, as indicated in Figure 11b. Hyperspectral images were taken and matched to the spectral libraries to map the presence and location of QD1− and QD1+ in COCs (**b**, **c**, respectively) and follicle sections (**e**, **f**, respectively). *Red arrows* indicate fluorescence signals in pseudo-red color. No fluorescence signals were seen in the controls (**a**, **d**).

This novel nano-scale imaging technology was used to further confirm the QD labeling of cells and map their localization. The hyperspectral fluorescence imaging combines digital image capturing and conventional spectroscopy to generate spectral signatures as a function of wavelength that characterizes samples. Samples without labeling (negative control) showed no fluorescence signals (Figures 11a,  12a, d), while small aliquots of QD suspensions and samples labeled with QD revealed reference spectral libraries with peak emission of or near 655 nm (Figure 11b), corresponding to the emission wavelength of the QD used in this study.

The fluorescence signal of QD1− was mostly detected in the sperm mid-piece while those from QD1+ were stronger and essentially located in the sperm head (Figure 11d, f). These results corroborated with those of the confocal microscope. Similar fluorescence intensities were observed with the cumulus-oocyte complexes or COCs (Figure 12b, c) and follicles (Figure 12e, f). In both sample types, the QD1+ fluorescence remained stronger than that of QD1−. The fluorescence signal ratios (QD1+/QD1−) were increased to approximately 1.6×, in spermatozoa (Figure 11f, d) and 9.3×, in COCs (Figure 12b, c).

Both COCs and follicle hyperspectral fluorescence image intensities matched the bioluminescence data. The application of this novel hyperspectral imaging validates the traditional microscope and confocal fluorescence technologies. The current study represents, to the best of our knowledge, the first report on mammalian reproductive cells that combines both nanotechnology and hyperspectral fluorescence for bio-imaging. The application of the hyperspectral fluorescence imaging is new in the reproductive field, but this technology has been applied in various research fields of biomedicine [52], including cancer and Parkinson’s disease diagnoses [53, 54] and tissue characterization [55].

## Conclusions

The current study uses various settings (in vitro, in situ, *and* ex vivo) to assess the effectiveness of a new nanotechnology-based imaging approach for mammalian reproductive cells. We combined traditional and novel imaging techniques to validate the nano-based labeling. The proposed-imaging technology offers the possibility for minimal invasive tracking of reproductive cells in their physiological environments, with the possibility to measure changes in cellular and molecular events that affect mammalian gamete quality. However, nanoparticle uptake by ovarian follicular cells and subsequent bio-imaging still need optimization in further experiments. Furthermore, the study confirms the presence of plasminogen protein in pig oocytes, while revealing its detection in mature spermatozoa. This immunopositive reactivity allows for further noninvasive and functional analyses of the influential range of plasminogen during the fertilization success and outcomes.

## Additional files

Additional file 1:
**Figure S1.** Dynamic Light Scattering (DSL) size measurements. Self-illuminating bioluminescent resonance electronic transfer quantum dots emitting at 655 nm (BRET-QD) and linked to nona-Arginine cell-penetrating peptides (R9) were functionalized (QD+) or not (QD−) with plasminogen antibody. Aliquots of each sample were dispersed in the incubation medium (PBS, pH 7.4) and submitted to the zetaPALS operated at 659 nm wavelength for DSL measurements at 37°C after 5 min equilibration. Monomer and aggregate sizes are indicated in black and blue colors, respectively. Additional file 2:
**Figure S2.** Transmission Electron Microscope imaging of labeled spermatozoa. Cross-section heads of spermatozoa labeled with functionalized (blue frame) and non-functionalized (black frame) QD-BRET are shown. Arrows indicate the localization of quantum dot (QD) core and higher accumulation can be seen in functionalized, compared to non-functionalized sections. Additional file 3:
** Figure S3.** Laser confocal microscope imaging of labeled cumulus-oocyte complexes and spermatozoa. The black frame regroups cumulus-oocyte complexes labeled with anti-human plasminogen antibody before (B) and after (C) in vitro maturation, while blue frame indicates spermatozoa labeled with the same antibody (E) and counterstained with DAPI for nuclei visualization (F). Micrographs A and D correspond to samples that were incubated without anti-plasminogen and served as negative controls. Arrow and arrow heads indicate the oocyte and the cumulus cells, respectively.

## Abbreviations

FITC-PSA: fluoresceinisocyanate conjugate-*Pisum sativum* agglutinin (pea)
BSA: bovine serum albumin
PBS: phosphate buffered solution

## References

[1] Kölle S, Reese S, Kummer W (2010). New aspects of gamete transport, fertilization, and embryonic development in the oviduct gained by means of live cell imaging. Theriogenology.

[2] Druart X, Cognie J, Baril G, Clement F, Dacheux JL, Gatti JL (2011). In vivo imaging of ram spermatozoa in the ewe genital tract using fibered confocal microscopy. Gynecol Obstet Fertil.

[3] Trottmann M, Stepp H, Sroka R, Heide M, Liedl B, Reese S (2014). Probe-based confocal laser endomicroscopy (pCLE) - a new imaging technique for in situ localization of spermatozoa. J Biophotonics.

[4] Resch-Genger U, Grabolle M, Cavaliere-Jaricot S, Nitschke R, Nann T (2008). Quantum dots versus organic dyes as fluorescent labels. Nat Meth.

[5] Feugang JM, Youngblood RC, Greene JM, Fahad AS, Monroe WA, Willard ST (2012). Application of quantum dot nanoparticles for potential non-invasive bio-imaging of mammalian spermatozoa. J Nanobiotechnol.

[6] So MK, Xu C, Loening AM, Gambhir SS, Rao J (2006). Self-illuminating quantum dot conjugates for in vivo imaging. Nat Biotechnol.

[7] Alivisatos AP, Gu W, Larabell C (2005). Quantum dots as cellular probes. Annu Rev Biomed Eng.

[8] Bakalova R, Zhelev Z, Kokuryo D, Spasov L, Aoki I, Saga T (2011). Chemical nature and structure of organic coating of quantum dots is crucial for their application in imaging diagnostics. Int J Nanomed.

[9] Medintz IL, Uyeda HT, Goldman ER, Mattoussi H (2005). Quantum dot bioconjugates for imaging, labelling and sensing. Nat Mater.

[10] Pinaud F, Michalet X, Bentolila LA, Tsay JM, Doose S, Li JJ (2006). Advances in fluorescence imaging with quantum dot bio-probes. Biomaterials.

[11] Law WC, Yong KT, Roy I, Ding H, Hu R, Zhao W (2009). Aqueous-phase synthesis of highly luminescent CdTe/ZnTe Core/Shell quantum dots optimized for targeted bioimaging. Small.

[12] Chen M, Yin M (2014). Design and development of fluorescent nanostructures for bioimaging. Top Issue Biorelated Polym.

[13] Zhang LW, Monteiro-Riviere NA (2009). Mechanisms of quantum dot nanoparticle cellular uptake. Toxicol Sci.

[14] Wang Y, Hu R, Lin G, Roy I, Yong KT (2013). Functionalized quantum dots for biosensing and bioimaging and concerns on toxicity. ACS Appl Mater Interfaces.

[15] Iyer G, Michalet X, Chang YP, Weiss S (2010). Tracking single proteins in live cells using single-chain antibody fragment-fluorescent quantum dot affinity pair. Methods Enzymol.

[16] Michalet X, Pinaud FF, Bentolila LA, Tsay JM, Doose S, Li JJ (2005). Quantum dots for live cells, in vivo imaging, and diagnostics. Science.

[17] Smith AM, Duan H, Mohs AM, Nie S (2008). Bioconjugated quantum dots for in vivo molecular and cellular imaging. Adv Drug Deliv Rev.

[18] Shi L, Rosenzweig N, Rosenzweig Z (2007). Luminescent quantum dots fluorescence resonance energy transfer-based probes for enzymatic activity and enzyme inhibitors. Anal Chem.

[19] Zhang W, Chen G, Wang J, Ye BC, Zhong X (2009). Design and synthesis of highly luminescent near-infrared-emitting water-soluble CdTe/CdSe/ZnS Core/Shell/Shell quantum dots. Inorg Chem.

[20] Quiñones GA, Miller SC, Bhattacharyya S, Sobek D, Stephan JP (2012). Ultrasensitive detection of cellular protein interactions using bioluminescence resonance energy transfer quantum dot-based nanoprobes. J Cell Biochem.

[21] Deliolanis NC, Kasmieh R, Wurdinger T, Tannous BA, Shah K, Ntziachristos V (2008). Performance of the red-shifted fluorescent proteins in deep-tissue molecular imaging applications. J Biomed Optics.

[22] Coy P, Jimenez-Movilla M, Garcia-Vazquez FA, Mondejar I, Grullon L, Romar R (2012). Oocytes use the plasminogen-plasmin system to remove supernumerary spermatozoa. Hum Reprod.

[23] Mondejar I, Grullon LA, Garcia-Vazquez FA, Romar R, Coy P (2012). Fertilization outcome could be regulated by binding of oviductal plasminogen to oocytes and by releasing of plasminogen activators during interplay between gametes. Fertil Steril.

[24] Grullon LA, Gadea J, Mondejar I, Matas C, Romar R, Coy P (2013). How is plasminogen/plasmin system contributing to regulate sperm entry into the oocyte?. Reprod Sci.

[25] Feugang JM, Greene JM, Willard ST, Ryan PL (2011). In vitro effects of relaxin on gene expression in porcine cumulus-oocyte complexes and developing embryos. Reprod Biol Endocrinol.

[26] Wu J, Emery BR, Carrell DT (2001). In vitro growth, maturation, fertilization, and embryonic development of oocytes from porcine preantral follicles. Biol Reprod.

[27] Feugang JM, Rodríguez-Muñoz JC, Dillard DS, Crenshaw MA, Willard ST, Ryan PL (2015). Beneficial effects of relaxin on motility characteristics of stored boar spermatozoa. Reprod Biol Endocrinol.

[28] Weissleder R (2001). A clearer vision for in vivo imaging. Nat Biotechnol.

[29] Frangioni JV (2003). In vivo near-infrared fluorescence imaging. Curr Opin Chem Biol.

[30] Kosaka N, Mitsunaga M, Bhattacharyya S, Miller SC, Choyke PL, Kobayashi H (2011). Self-illuminating in vivo lymphatic imaging using a bioluminescence resonance energy transfer quantum dot nano-particle. Contrast Media Mol Imaging.

[31] Saksela O, Vihko KK (1986). Local synthesis of plasminogen by the seminiferous tubules of the testis. FEBS Lett.

[32] Zhang L, Seiffert D, Fowler BJ, Jenkins GR, Thinnes TC, Loskutoff DJ (2002). Plasminogen has a broad extrahepatic distribution. Thromb Haemost.

[33] Rijken DC, Wijngaards G, Welbergen J (1981). Immunological characterization of plasminogen activator activities in human tissues and body fluids. J Lab Clin Med.

[34] Finlay TH, Katz J, Kirsch L, Levitz M, Nathoo SA, Seiler S (1983). Estrogen-stimulated uptake of plasminogen by the mouse uterus. Endocrinology.

[35] Huarte J, Vassalli J-D, Belin D, Sakkas D (1993). Involvement of the plasminogen activator/plasmin proteolytic cascade in fertilization. Dev Biol.

[36] Choi YJ, Uhm SJ, Song SJ, Song H, Park JK, Kim T (2008). Cytochrome c upregulation during capacitation and spontaneous acrosome reaction determines the fate of pig sperm cells: linking proteome analysis. J Reprod Dev.

[37] Feugang JM, Pendarvis K, Crenshaw M, Willard ST, Ryan PL (2010). Hight-throughput proteomics assessment of frozen-thawed boar spermatozoa. Reprod Fertil Dev.

[38] Zimmerman SW, Manandhar G, Yi Y-J, Gupta SK, Sutovsky M, Odhiambo JF (2011). Sperm proteasomes degrade sperm receptor on the egg zona pellucida during mammalian fertilization. PLoS One.

[39] Lefievre L, Bedu-Addo K, Conner SJ, Machado-Oliveira GSM, Chen Y, Kirkman-Brown JC (2007). Counting sperm does not add up any more: time for a new equation?. Reproduction.

[40] Pittayanon R, Rerknimitr R, Wisedopas N, Khemnark S, Thanapirom K, Thienchanachaiya P (2012). The learning curve of gastric intestinal metaplasia interpretation on the images obtained by probe-based confocal laser endomicroscopy. Diagn Ther Endosc.

[41] Shahid MW, Buchner AM, Coron E, Woodward TA, Raimondo M, Dekker E (2011). Diagnostic accuracy of probe-based confocal laser endomicroscopy in detecting residual colorectal neoplasia after EMR: a prospective study. Gastrointest Endosc.

[42] Yserbyt J, Dooms C, Ninane V, Decramer M, Verleden G (2013). Perspectives using probe-based confocal laser endomicroscopy of the respiratory tract. Swiss Med Wkly.

[43] Druart X, Cognie J, Baril G, Clement F, Dacheux JL, Gatti JL (2009). In vivo imaging of in situ motility of fresh and liquid stored ram spermatozoa in the ewe genital tract. Reproduction.

[44] Graham JK (2001). Assessment of sperm quality: a flow cytometric approach. Anim Reprod Sci.

[45] Barchanski A, Taylor U, Sajti CL, Gamrad L, Kues WA, Rath D (2015). Bioconjugated gold nanoparticles penetrate into spermatozoa depending on plasma membrane status. J Biomed Nanotechnol.

[46] Taylor U, Barchanski A, Petersen S, Kues WA, Baulain U, Gamrad L (2014). Gold nanoparticles interfere with sperm functionality by membrane adsorption without penetration. Nanotoxicology.

[47] Hunter M (1998). Follicular factors regulating oocyte maturation and quality. Hum Fertil (Camb).

[48] Moor R, Lee C, Dai Y, Fulka J (1996). Antral follicles confer developmental competence on oocytes. Zygote.

[49] Jung SY, Willard S (2014). Quantitative bioluminescence imaging of transgene expression in intact porcine antral follicles in vitro. Reprod Biol Endocrinol.

[50] Tiedemann D, Taylor U, Rehbock C, Jakobi J, Klein S, Kues WA (2014). Reprotoxicity of gold, silver, and gold-silver alloy nanoparticles on mammalian gametes. Analyst.

[51] Barkalina N, Jones C, Kashir J, Coote S, Huang X, Morrison R (2014). Effects of mesoporous silica nanoparticles upon the function of mammalian sperm in vitro. Nanomed Nanotechnol Biol Med.

[52] Carrasco O, Gomez RB, Chainani A, Roper WE (2003) Hyperspectral imaging applied to medical diagnoses and food safety. In: AeroSense 2003. International Society for Optics and Photonics, pp 215–221

[53] Lu G, Halig L, Wang D, Qin X, Chen ZG, Fei B (2014). Spectral-spatial classification for noninvasive cancer detection using hyperspectral imaging. J Biomed Optics.

[54] Oh ES, Heo C, Kim JS, Suh M, Lee YH, Kim JM (2014). Hyperspectral fluorescence imaging for cellular iron mapping in the in vitro model of Parkinson’s disease. J Biomed Optics.

[55] Zuzak KJ, Schaeberle MD, Lewis EN, Levin IW (2002). Visible reflectance hyperspectral imaging: characterization of a noninvasive, in vivo system for determining tissue perfusion. Anal Chem.

