# Supplementary figures and images for: Self-illuminating quantum dots for non-invasive bioluminescence imaging of mammalian gametes
Source: J Nanobiotechnology. 2015 Jun 4;13:38. doi: 10.1186/s12951-015-0097-1 (PMC4455054; doi:10.1186/s12951-015-0097-1)

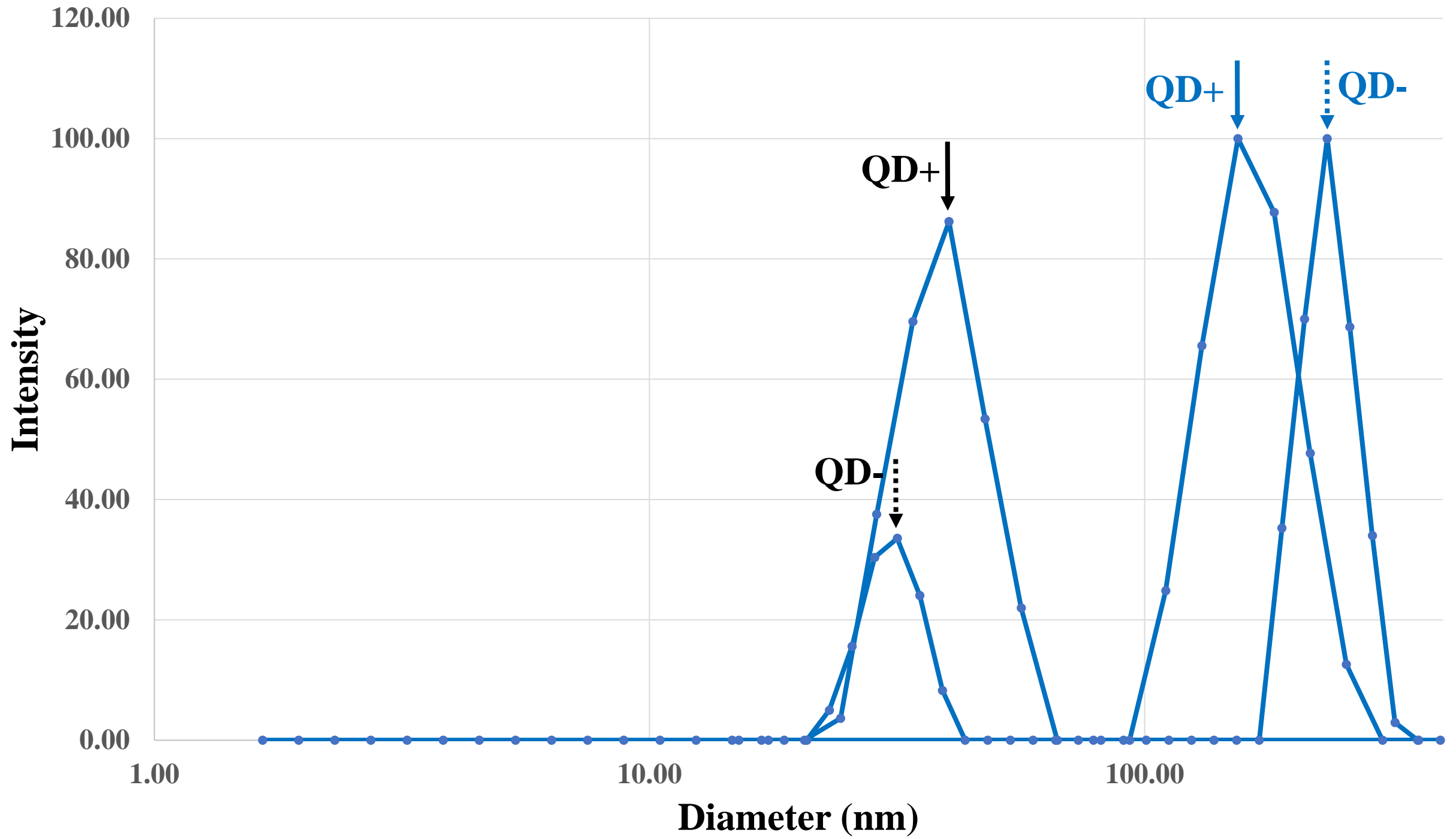

Supplement: Additional file 1: — Figure S1. Dynamic Light Scattering (DSL) size measurements. Self-illuminating bioluminescent resonance electronic transfer quantum dots emitting at 655 nm (BRET-QD) and linked to nona-Arginine cell-penetrating peptides (R9) were functionalized (QD+) or not (QD−) with plasminogen antibody. Aliquots of each sample were dispersed in the incubation medium (PBS, pH 7.4) and submitted to the zetaPALS operated at 659 nm wavelength for DSL measurements at 37°C after 5 min equilibration. Monomer and aggregate sizes are indicated in black and blue colors, respectively. [file 12951_2015_97_MOESM1_ESM.pdf]

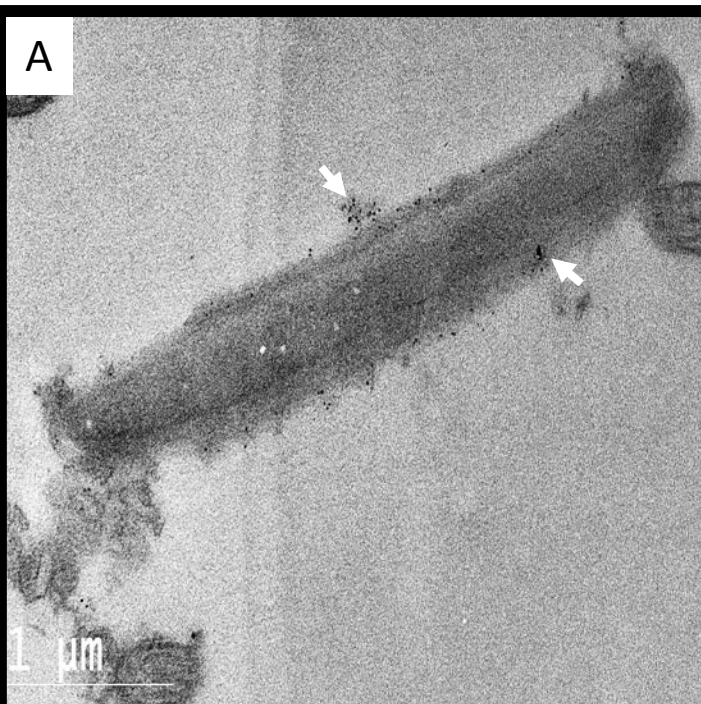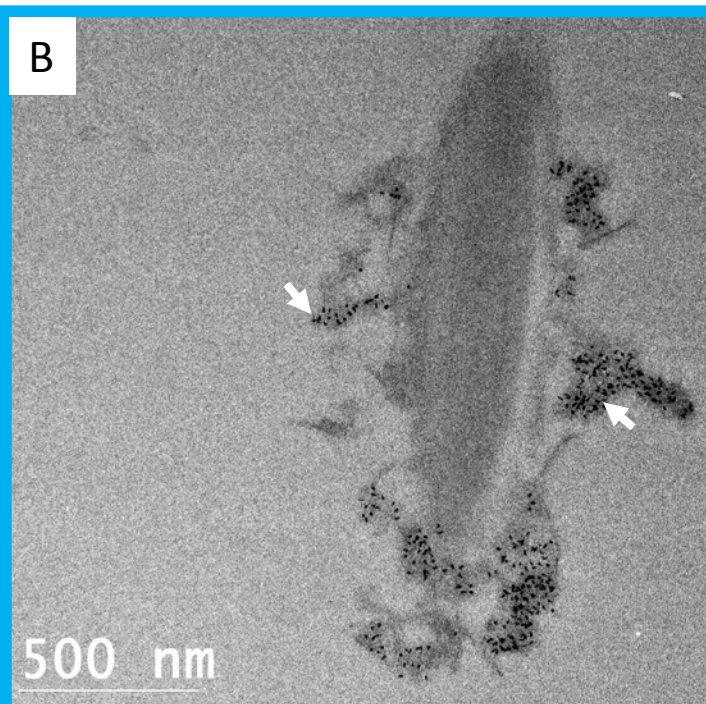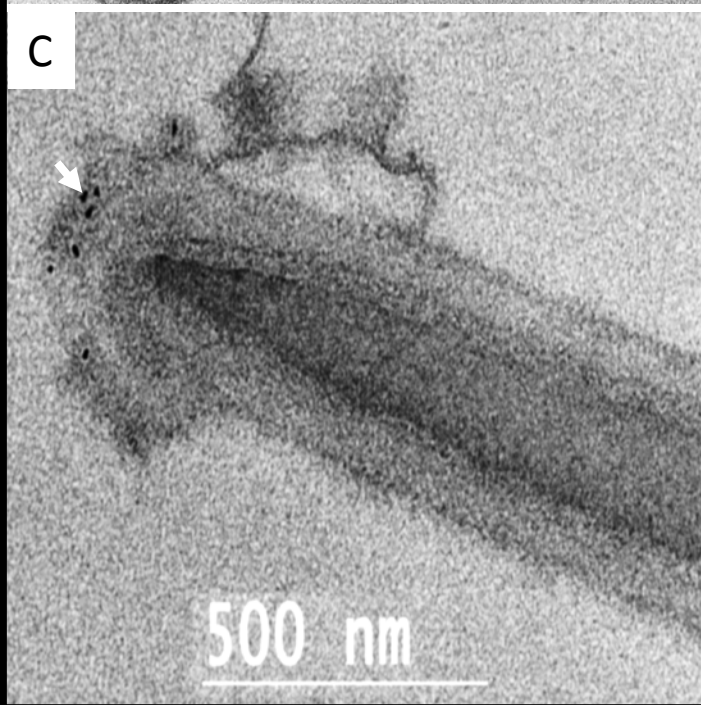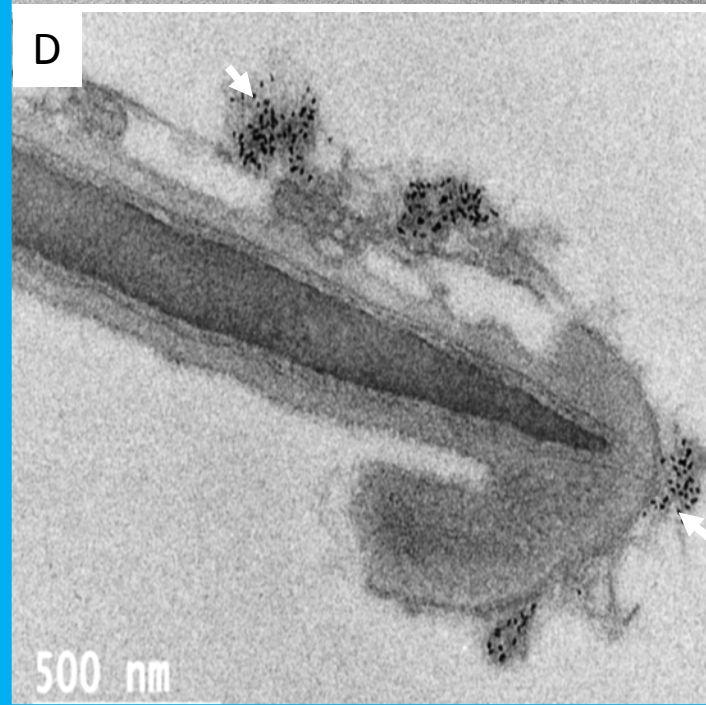

Supplement: Additional file 2: — Figure S2. Transmission Electron Microscope imaging of labeled spermatozoa. Cross-section heads of spermatozoa labeled with functionalized (blue frame) and non-functionalized (black frame) QD-BRET are shown. Arrows indicate the localization of quantum dot (QD) core and higher accumulation can be seen in functionalized, compared to non-functionalized sections. [file 12951_2015_97_MOESM2_ESM.pdf]

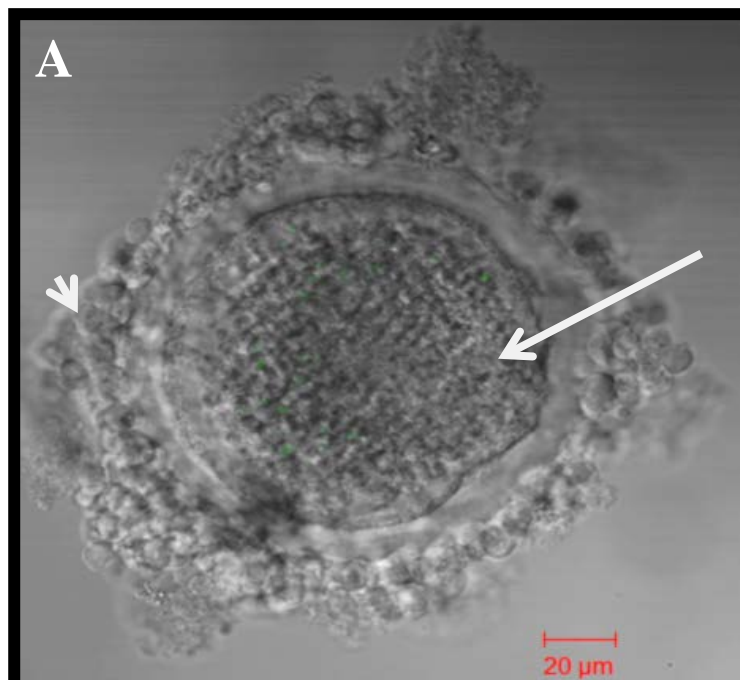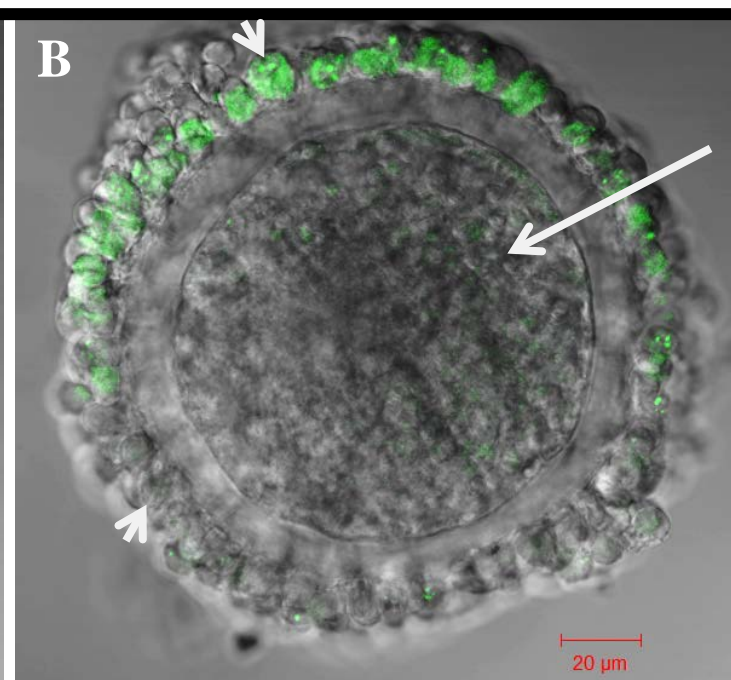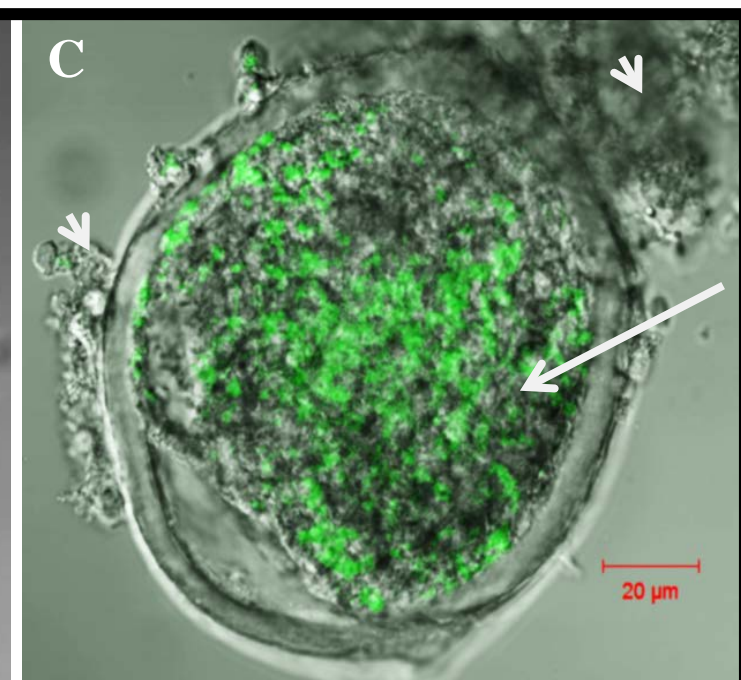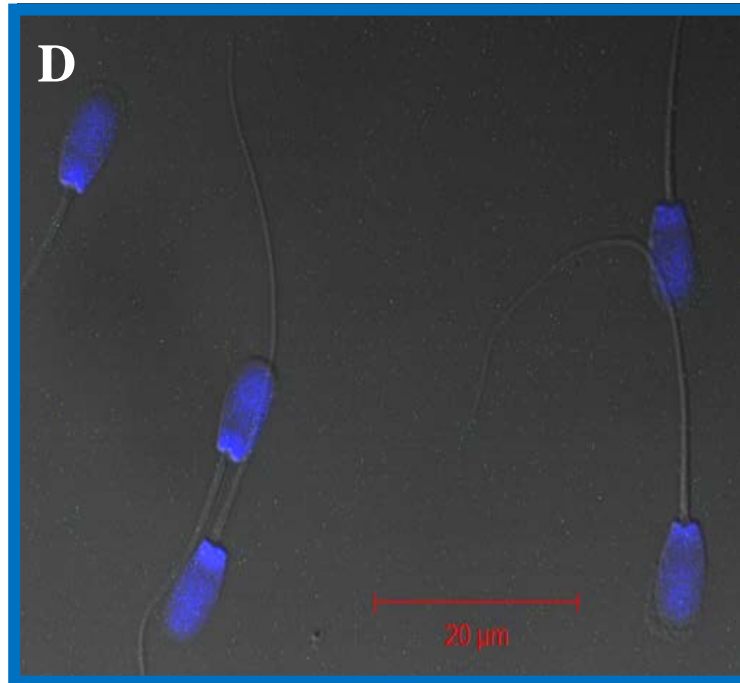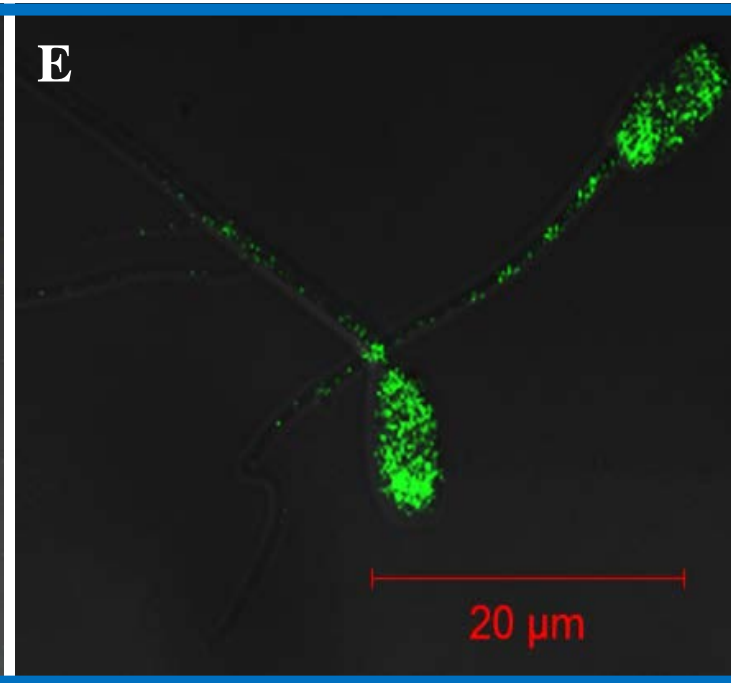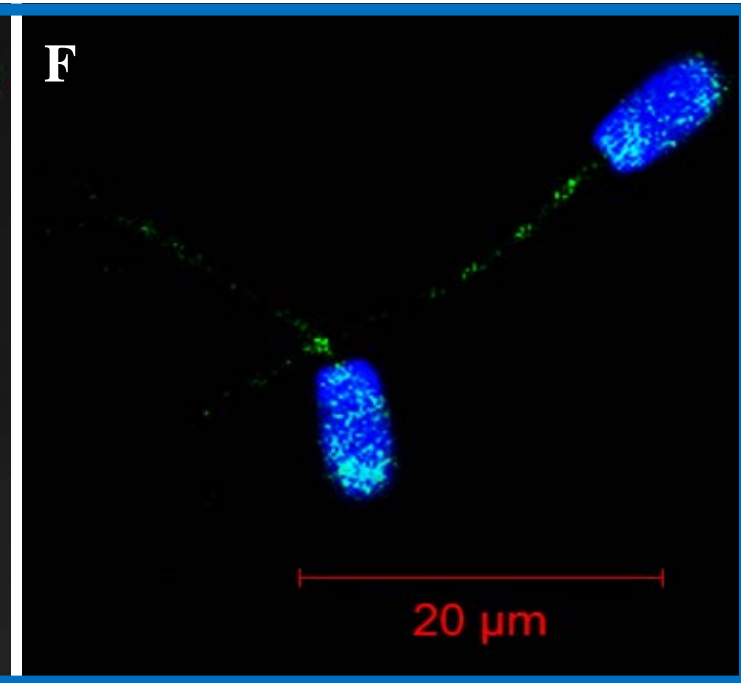

Supplement: Additional file 3: — Figure S3. Laser confocal microscope imaging of labeled cumulus-oocyte complexes and spermatozoa. The black frame regroups cumulus-oocyte complexes labeled with anti-human plasminogen antibody before (B) and after (C) in vitro maturation, while blue frame indicates spermatozoa labeled with the same antibody (E) and counterstained with DAPI for nuclei visualization (F). Micrographs A and D correspond to samples that were incubated without anti-plasminogen and served as negative controls. Arrow and arrow heads indicate the oocyte and the cumulus cells, respectively. [file 12951_2015_97_MOESM3_ESM.pdf]
